# Gastrodin alleviates high fructose-induced podocyte mitochondria-mediated apoptosis by inhibiting NLRP6 to facilitate TRIM7-triggered *Bok* mRNA degradation

**DOI:** 10.7150/ijbs.120307

**Published:** 2026-01-01

**Authors:** Hong Ding, Wen-Xuan Wang, Qiong-Dan Liang, Chuan-Feng Tang, Tang-Di Xu, Zi-An Miao, Bang-Xing Han, Ling-Dong Kong

**Affiliations:** 1 State Key Laboratory of Pharmaceutical Biotechnology, Institute of Chinese Medicine, Nanjing Drum Tower Hospital, School of Life Sciences, Nanjing University, Nanjing, China.; 2 Traditional Chinese Medicine Institute of Anhui Dabie Mountain, West Anhui University, Luan 237012, China.

**Keywords:** NLRP6, podocyte, mitochondria-mediated apoptosis, BOK, gastrodin

## Abstract

Mitochondria-mediated apoptosis is the key determinant of glomerular podocyte injury. NOD-like receptor family pyrin domain proteins (NLRPs) are aberrant in clinical kidney diseases, but the role in podocyte mitochondrial dysfunction is unclear. Here, we first observed only NLRP6 expression change in nephrotic syndrome patients with proteinuria. Next, we found that mouse glomerular podocyte NLRP6 expression was increased in high fructose-induced proteinuria with mitochondria-mediated apoptosis. Importantly, *Nlrp6* deficiency ameliorated these disturbances in mice. NLRP6 downregulation inhibited podocyte mitochondrial outer membrane permeabilization (MOMP)-associated apoptosis via suppressing B-cell lymphoma 2-related ovarian killer (BOK) under high fructose stimulation. However, high NLRP6 expression blocked the binding of Tripartite motif-containing protein 7 (TRIM7) with *Bok* mRNA 3' untranslated region, decreased mRNA decay, and thereby downregulated antioxidant protein family with sequence similarity 213, member A (FAM213A), resulting in mitochondria-mediated apoptosis in high fructose-exposed podocytes. A nephroprotective agent gastrodin was found to decrease NLRP6 and relieve mitochondria-mediated apoptosis caused by high fructose, possibly through promoting TRIM7-driven *Bok* mRNA degradation and FAM213A antioxidant effect. This study uncovered that high NLRP6 expression-driven mitochondria-mediated apoptosis could participate in podocyte injury and the suppression of NLRP6 by gastrodin may be an attractive therapeutic approach for podocyte injury.

## Introduction

Podocyte, as a terminal epithelial cell, is indispensable to the maintenance of normal glomerular filtration. Podocyte destruction induces proteinuria in common renal disease patients in clinical trials 1. Current treatments aiming at avoiding or decreasing podocyte injury are usually nonspecific immunosuppressants or anti-inflammatory agents, which come with undesirable adverse side effects 2. The underlying mechanisms of podocyte injury are still poorly understood.

The Western dietary pattern containing high fructose is a recognized risk factor for metabolic syndrome and chronic kidney disease 3. Our prior study showed that excessive fructose intake produced glomerular podocyte apoptosis-related proteinuria and promoted podocyte mitochondrial metabolic reprogramming in rats 4, 5. In fact, mitochondrial dysfunction is the leading cause of podocyte apoptosis 6. Thus, targeted intervention of mitochondrial dysfunction and associated apoptosis could offer a novel therapeutic approach to prevent glomerular podocyte injury induced by high fructose.

Mitochondrial outer membrane permeabilization (MOMP) is defined as the initial process of mitochondrial or intrinsic pathway apoptosis, causing Cytochrome C (Cyto C) leakage, Caspase activation, and cell apoptosis 7. B-cell lymphoma 2 (Bcl-2)-related ovarian killer (BOK) at high expression level directs apoptosis-associated MOMP via formatting a mixture of oligomers to open apoptotic pores in human colon cancer cells lacking Bcl-2 antagonist killer (BAK) and Bcl-2 associated X (BAX) 8. Thus, under high fructose exposure, whether BOK-activated MOMP drives podocyte mitochondria-mediated apoptosis is worth clarifying. Reactive oxygen species (ROS) accumulation is frequently detected in podocyte apoptosis. In high fructose-exposed podocytes, excess ROS reduces the mitochondrial membrane potential and injures mitochondrial structure, causing podocyte apoptosis 9. Family with sequence similarity 213, member A (FAM213A) as a dual antioxidant protein protects monocyte cells from hydrogen peroxide-induced oxidative stress 10. Our earlier research showed that FAM213A expression was decreased in glomeruli of high fructose-fed rats, as analyzed through proteomics and transcriptomics 5. The inhibition of FAM213A-mediated oxidative stress may be a feasible strategy to relieve podocyte mitochondria-mediated apoptosis under high fructose stimulation.

Gastrodin, an extract from the Chinese Pharmacopoeia-listed herb *Gastrodia elata* Blume, possesses mitochondria-protecting property 11. Gastrodin relieves respiratory suppression and cell viability in hydrogen peroxide-induced oxidative damage of mouse hippocampal neuronal cells 12. This compound protects the kidney from inflammation, fibrosis, ferroptosis, and apoptosis through antioxidant signaling pathways 13-15. Therefore, the beneficial effects of gastrodin on podocyte mitochondria-mediated apoptosis warrant further investigation.

The NOD-like receptor family pyrin domain proteins (NLRPs) primarily drive inflammasome assembly and activation, which can mediate mitochondrial function 16. Here, we first observed abnormal expression of *Nlrp6* and *Nlrp12* in diverse clinical nephropathy samples, and found that only *Nlrp6* expression was changed in nephrotic syndrome patients, displaying a significant positive correlation with proteinuria. NLRP6 is reported to modulate immune responses against bacterial infections in host defense 17, regulate mucus secretion and antimicrobial peptide formation 18, 19. It also restrains the progression of non-alcoholic steatohepatitis 20, promotes repair of peripheral nerve injury 21, and relieves stress-induced depression 22. These observations suggest NLRP6 as a key factor of tissue homeostasis across multiple organs. However, its specific function and the underlying mechanism in glomerular podocyte injury, remain entirely unexplored. Our study showed that NLRP6 as a major promoter of podocyte mitochondria-mediated apoptosis injury caused by high fructose. NLRP6 inhibited Tripartite motif-containing protein 7 (TRIM7)-mediated *Bok* mRNA decay and downregulated FAM213A to increase ROS accumulation, which ultimately induced mitochondria-mediated apoptosis during high fructose exposure. Gastrodin inhibited podocyte mitochondria-mediated apoptosis by suppressing NLRP6 expression to increase TRIM7-triggered* Bok* mRNA degradation as well as FAM213A antioxidant capacity. Our study provides a creative mechanism of high fructose-caused podocyte mitochondria-mediated apoptosis and improves the knowledge of therapeutic strategy for NLRP6 inhibition by gastrodin.

## Materials and Methods

### Clinical data analysis

The data were obtained from RNA sequencing (RNA-seq) profiles of blood transcriptomic samples in the Gene Expression Omnibus (GEO) database (https://www.ncbi.nlm.nih.gov/geo/), comprising 5 diabetic nephropathy (DN) patients and 5 healthy volunteers (GSE154881). Differential expression genes (DEGs) were performed using the “Limma” R package. Statistically significant DEGs were identified under dual thresholds of |log_2_Foldchange| > 1.0 and *P* - value < 0.05. Volcano plot was visualized using the “ggplot2” R package. In addition, we obtained *Nlrps* mRNA expression in healthy human kidney tissues from the Genotype-Tissue Expression (GTEx) database (https://www.gtexportal.org/) and used the “recount3” R package to analyze the expression in the renal cortex (*n* = 94) and medulla (*n* = 4). Box plots were visualized using the “ggplot2” R package.

Transcriptomic data of human renal biopsy specimens were obtained from Nephroseq (https://www.nephroseq.org/). *Nlrp6* expression was from RNA-seq analysis of the Nakagawa Chronic kidney disease (CKD) kidney dataset, including 53 renal biopsies from 48 individuals with CKD and 5 healthy living donors. Affymetrix human microarray results of Sampson Nephrotic syndrome glomeruli datasets were from 6 minimal change disease (MCD) samples, 8 membranous glomerulonephropathy samples, and 6 other nephrotic syndrome samples. Correlation of expression to proteinuria was performed using RNA-seq data of Mariani nephrotic syndrome glomeruli dataset from 35 nephrotic proteinuria samples.

### Animals and procedures

C57BL/6JGpt male mice (RRID: IMSR_GPT: N000013) were acquired from GemPharmatech Co., Ltd. and housed in the Animal Research Center's specific pathogen-free facility at Nanjing University. All experimental protocols and husbandry for animal studies were authorized by the ethics committee of the Nanjing University (Approval No: IACUC-2006017). All animal experiments were conducted in compliance with the European Union Directive 2010/63/EU.

*Nlrp6* knockout (*Nlrp6^-/-^*) mice were generated via CRISPR-Cas9 genome editing technology, per a work described previously 23. We constructed an *Nlrp6^-/-^* mice by deleting exons 1-8, resulting in the absence of *Nlrp6* expression. Genomic DNA extracted from mouse tails by qPCR was used for mouse genotyping.

For high fructose diet (HFrD)-fed mouse model, using a random number table method, four groups of 6-week-old wild type (WT) and *Nlrp6^-/-^* mice were randomly assigned: WT-normal chow (*n* = 15), WT-HFrD (*n* = 15), *Nlrp6^-/-^*-normal chow (*n* = 15), *Nlrp6^-/-^-*HFrD (*n* = 15). HFrD group were fed with HFrD (60%, TD.89247, Harlan Teklad) for 12 weeks, while normal chow group were fed no-fructose control diet (TD.05075, Harlan Teklad). Mice were sacrificed after HFrD feeding procedure ended. The duration of each dietary challenge was also noted in corresponding figure legends.

For drug intervention, 75 mice were initially assigned into two groups: the HFrD group (*n* = 60) received 12 weeks of feeding with 60% HFrD, while control group (*n* = 15) received a no-fructose control diet. After 4-week HFrD acclimatization, control mice received daily vehicle placebo (0.9% saline) via intragastric gavage for 8 weeks. The remaining HFrD-fed mice were subsequently randomized (*n* = 15 per group) to undergo an 8-week daily gavage with either the vehicle or gastrodin (G299059, Aladdin; PubChem CID: 115067) at 25, 50, and 100 mg/kg. All mice were sacrificed following this intervention period.

Throughout the course of the experiment, weekly record of body weight was collected. Weekly urine samples were gathered to check albuminuria by the metabolic cage. Serum and urine samples were obtained for biochemical testing at the end of the study. Following euthanasia via pentobarbital sodium anesthesia (40 mg/kg) and cervical dislocation, glomeruli were isolated for molecular experimental analysis, and sections of kidney tissue were taken for histological analysis.

### Isolation of mouse glomeruli

As previously described 24, Dynabead perfusion was used to isolate mouse glomeruli. To summarize, the renal cortex was finely chopped into pieces and subjected to an enzymatic digestion buffer containing 10 mg/mL of collagenase A (C6885, Sigma-Aldrich), 10 mg/mL of protease (P6911, Sigma-Aldrich), and 100 U/mL of DNase I (D5025, Sigma-Aldrich) in Hanks balanced salt solution (HBSS) for a duration of 15 minutes (min) at 37 °C. The decomposed tissue was recovered by centrifuging at 250 *g* at 4 °C for 10 min after being gently squeezed through a 100 μm filter and periodically flushed with HBSS. After reconstituting the pellet in HBSS, glomeruli were collected by magnetic particle concentrator and cleaned with HBSS. Through microscopy, glomeruli's purity was confirmed.

### RNAscope *in situ* hybridization

Following an established protocol 23, the fresh frozen kidney sections (10 μm) were used in this procedure. After the *in-situ* hybridization staining was finished, we proceeded with the standard immunofluorescence staining procedure to co-stain with antibodies.

### Biochemical analysis of serum and urine samples

Commercially available biochemical kits were used to assess levels of serum uric acid (C012-2-1), creatinine (C011-1-1), urea nitrogen (C013-2-1), urine creatinine (C011-1-1), as well as urine protein (C035-2-1). The commercially available ELISA kit (H127-1-2) was used to assay urine albumin, as directed by the manufacturer. The kits mentioned above were obtained from JianCheng Bioengineering Institute.

### Histological analysis

The mouse kidney was preserved in 4% paraformaldehyde (PFA), encased in paraffin, and sectioned into 4 µm-thick sections. These were then processed for Periodic acid-Schiff (PAS), Hematoxylin-Eosin (HE), and Masson staining, utilizing the manufacturer's recommended protocols by using corresponding staining kits, respectively. The slices underwent examination and photographed with a Pannoramic MIDI II (3DHISTECH Ltd.). The Image J software (National Institutes of Health) was used to quantify mesangial expansion as a percentage of glomeruli having mesangial positive areas.

### Transmission electron microscopy (TEM)

Microstructure of mouse glomeruli and podocytes was analyzed through standard transmission electron microscopy. Following usual protocol, the fresh kidney was fixed for 2 hours (h) with the 2.5% glutaraldehyde mixture, then it was cleaned, dried, and placed into resin. Samples were examined and captured photographs by electron microscope (HT7700, HITACHI). Using Image J software (National Institutes of Health), the foot process width, glomerular basement membrane (GBM) thickness, and number of foot processes per μm of GBM were determined. From each mouse, two glomeruli were chosen at random, and inside each glomerulus, 10 electron micrographs were collected. More than 70 mitochondria from each set of three mice were analyzed for mitochondrial research.

### Cell culture, treatment, and transfection

Mouse podocyte clone-5 cells (MPC5) were obtained from Shanghai Fuheng Biotechnology Co., Ltd, and maintained in RPMI 1640 medium supplemented with 10% fetal bovine serum, 100 U/mL penicillin, and 0.1 mg/mL streptomycin at 37 °C with 5% CO_2_.

Human podocytes (HPCs) were presented by Dr. Zhi-Hong Liu at the Research Institute of Nephrology, Nanjing General Hospital of Nanjing Military Command, Nanjing, China, was utilized in this study. HPCs were cultured in RPMI-1640 medium supplemented with 10% fetal bovine serum, 100 U/mL penicillin, 0.1 mg/mL streptomycin, and recombinant interferon-γ (CAA31639, R&D) at 33 °C with 5% CO_2_, and differentiated at 37 °C without recombinant interferon-γ.

Human glomerular mesangial cells (HGMCs) and primary mouse glomerular endothelial cells (PMGECs) were obtained from Wuhan Pricella Biotechnology Co., Ltd. HGMCs were maintained in DMEM medium supplemented with 10% fetal bovine serum, 100 U/mL penicillin, and 0.1 mg/mL streptomycin at 37 °C with 5% CO_2_. PMGECs were maintained specialized medium for endothelial cells (CM-M063, Pricella) at 37 °C with 5% CO_2_. Human proximal tubule epithelial cells (HK-2) were obtained from Cell Bank of Chinese Academy of Science, supplied by ATCC in Wuhan University, and maintained in DMEM/F-12 medium supplemented with 10% fetal bovine serum, 100 U/mL penicillin, and 0.1 mg/mL streptomycin at 37 °C with 5% CO_2_.

Primary mouse podocytes (PMPCs) were obtained by culturing isolated glomeruli in RPMI 1640 medium in culture flasks coated with type I collagen. After 7 days of culture, PMPCs migrated out from the glomeruli. At 80% confluence, PMPCs were detached and subsequently passed through a 30 μm sieve to remove residual glomerular cores. Purified PMPCs were maintained in RPMI 1640 medium supplemented with 10% fetal bovine serum, 100 U/mL penicillin, and 0.1 mg/mL streptomycin at 37 °C with 5% CO_2_ for subsequent experiments.

Different stimuli were used in this study: (1) fructose (5 mM, 1286504, Sigma-Aldrich); (2) a protein synthesis inhibitor cycloheximide (CHX, 5 μg/mL, M4879, Abmole); (3) RNA synthesis inhibitor actinomycin D (Act D, 5 μg/mL, M4881, Abmole); (4) MitoTEMPO (10 μM, HY-112879, MCE); (5) gastrodin (25, 50, and 100 μM, HY-N0115, MedChemExpress).

For transfection, Lipofectamine 2000 reagent (11668-019, Invitrogen) was used to deliver short interfering RNA (siRNA) or plasmids into cells following the manufacturer's instructions. (1) MPC5 were transfected with siRNA-Negative control (siRNA-NC) or siRNA-*Nlrp6*, subsequently stimulated with 5 mM fructose or not. (2) MPC5 were transfected with siRNA-NC or siRNA-*Bok*, subsequently stimulated with 5 mM fructose or not. (3) MPC5 were transfected with vector or *Trim7-Flag*, subsequently stimulated with 5 mM fructose or not. (4) MPC5 were transfected with vector or *Trim7-Flag*, subsequently stimulated with 5 μg/mL CHX or not. (5) MPC5 were transfected with vector or *Trim7-Flag*, subsequently stimulated with 5 μg/mL Act D or not. (6) MPC5 were transfected with vector or *Nlrp6-Flag* or siRNA-*Bok*. (7) MPC5 were transfected with vector or *Nlrp6-Flag*, subsequently stimulated with 5 μg/mL CHX or not. (8) MPC5 were transfected with vector or *Nlrp6-Flag*, subsequently stimulated with 5 μg/mL Act D or not. (9) MPC5 were transfected with vector or *Bok-HA*, subsequently stimulated with 10 μM MitoTEMPO or not. (10) MPC5 were transfected with vector or *Fam213a-Flag*. (11) MPC5 were transfected with vector or *Bok-HA* or *Fam213a-Flag*. (12) MPC5 were transfected with vector or *Nlrp6-Flag*, subsequently stimulated with 5 mM fructose or 100 μM gastrodin. The sequences of the siRNAs used are shown in Table S1.

### Immunofluorescence (IF) staining

After 24 h of fixation in 4% PFA, the whole mouse kidney or kidney cortex was placed in O.C.T. compound, respectively, then cut it into 5 μm portions as frozen slices, respectively. On coverslips, HPCs, MPC5, and PMPCs were fixed in 4% PFA. Frozen slices or coverslips were cleaned with phosphate buffer saline, followed by permeabilization with blocking buffer. Slices were exposed to primary antibodies at 4 °C overnight, prior to a 1 h incubation with secondary antibodies at room temperature. We utilized 4',6-diamidino-2-phenylindole (DAPI, C1002, Beyotime) to stain nuclear. Confocal scanning microscope (Leica) or slide scanner (VS200, Olympus) was used to evaluate the samples and capture photographs. The intensity of protein fluorescence staining was measured by Image J software (National Institutes of Health). Table S2 provides a summary of the antibodies utilized in this investigation.

### Western blot analysis

Protein extracts from mouse glomeruli, MPC5, HPCs, PMPCs, HGMCs, PMGECs and HK-2 were homogenized in lysis buffer, followed by centrifugation at 12, 000 *g* for 15 min at 4 °C, respectively. The protein concentration of these samples was adjusted by the BCA kit (23227, Thermo), then added with loading buffer and boiled. Proteins in equal quantities from every sample were subjected to electrophoresis on 10% SDS-PAGE gels and electrotransferred to polyvinylidene fluoride membranes (IPVH100010, Millipore). Following a blocking step, the membranes probed with primary antibodies and secondary antibodies. Table S2 provided a summary of the antibodies utilized in this investigation.

Immunoreactive proteins were visualized using enhanced chemiluminescence (180-5001W, Tanon). An enhanced chemiluminescence system (Tanon) was then used to observe the signals and capture the photographs. Densitometry was used to quantify the immunoreactive blots using Image J software, standardizing the results to β-actin, expressing the results as fold changes in relation to control.

### RNA extraction and quantitative real-time (qRT)-PCR

RNA isolation of mouse glomeruli and MPC5 was conducted using the Trizol method (R401-01-AA, Vazyme), as recommended by the manufacturer. 1 μg of total RNA was produced with HiScript® II Q RT SuperMix (R222, Vazyme) to synthesize single-strand cDNA. Complementary DNA was amplified using ChamQTM SYBR® qPCR Master Mix (Q311, Vazyme) in a Bio-Rad PCR System. The expression of RNA was compared to that of β-actin. Table S3 displayed the primers that were used in this investigation.

### Apoptosis analysis

Using the fluorescein isothiocyanate-conjugated Annexin V and Propidium iodide (Annexin V-FITC/PI) staining kit (556547, BD) as directed, cell apoptosis was identified. The Attune NxT Acoustic Focusing Cytometer (Invitrogen) was used to assess apoptotic cells that had been incubated with probes for 15 min at room temperature. FlowJo 10.4 software (BD) was applied to analyze data.

Glomerular podocyte apoptosis was detected using a TUNEL assay kit (A112-03, Vazyme). Briefly, kidney frozen sections and cell climbing slides were fixed with 4% PFA, permeabilized with 0.2% Triton X-100, and washed with PBS, respectively. The samples were then incubated with the dUTP Mix at 37°C for 60 min, followed by PBS rinsing. Finally, the sections were mounted with an antifade medium containing DAPI and visualized under a fluorescence microscope.

### Isolation of glomeruli and podocyte mitochondria

Mitochondria from mouse glomeruli and podocytes were obtained by employing a mitochondrial extraction kit (SM0020, Solarbio), respectively. Following the lysis of isolated mitochondria in buffer, the protein concentration was adjusted via BCA kit (23227, Thermo).

### Mitochondrial membrane potential assay

Mitochondrial membrane potential levels of MPC5, PMPCs, and HPCs were detected with JC-10 probes (22204, AATbio) in accordance with instructions. 1 × 10^5^ cells were gathered and stained 3 µM JC-10 working solution for 30 min in a 37 °C, 5% CO_2_ incubator. After incubation, Attune NxT Acoustic Focusing Cytometer (Invitrogen) was used to record the fluorescence of samples, and FlowJo 10.4 software (BD) was used to analyze the ratio of red fluorescence (aggregated JC-10) to green fluorescence (monomeric JC-10), indicating changes in the ΔΨm.

### Oxygen consumption rate (OCR) measurement

Mitochondrial OCR was assessed using the XFe96 extracellular flux analyzer (Seahorse Bioscience) with the mitochondrial stress test kit (103015100, Agilent), following a previously established procedure. In brief, MPC5 cells were seeded into Seahorse microplates, cultured overnight, and then treated with various stimulations before measurement. Prior to assay, the cells were washed and equilibrated for 1 h in Seahorse XF DMEM base medium supplemented with 1 mM sodium pyruvate, 2 mM glutamine, and 10 mM glucose in a non-CO_2_ incubator at 37 °C. The mitochondrial stress test was then performed by the serial injection of oligomycin (1.5 µM), carbonyl cyanide 4-(trifluoromethoxy) phenylhydrazone (1 µM), and rotenone/antimycin A (0.5 µM) as final concentration. All measurements were conducted per the manufacturer's instructions and normalized to the cellular protein content determined by a BCA assay (23227, Thermo).

### Caspase 3 and Caspase 9 activity assay

Podocyte Caspase 3 activity level was assessed by the commercially available kit (C1168M, Beyotime). Activated Caspase3-positive cell level was assessed using Cleaved Caspase 3 (Asp175) antibody (Alexa Fluor ®488 Conjugate) (9669S, CST) by flow cytometry. 96-well plate-cultured podocytes and 6-well plate-cultured podocytes were stimulated with or without 5 mM fructose after being transfected with siRNA-*Nlrp6* or siRNA-NC. Caspase 3 activity in mouse glomeruli was evaluated using the commercially available kit (C1116, Beyotime) following manufacturer's specifications. For tissue analysis, 10 mg of mouse glomeruli were lysed, centrifuged, and transferred to the supernatant for Caspase 3 activity analysis.

Caspase 9 activity in podocytes and glomeruli were tested with the commercially available kit (C1158, Beyotime) in compliance with the manufacturer's guidelines. For cells, 6-well plates podocytes were stimulated with or without 5 mM fructose after being transfected with siRNA-*Nlrp6* or siRNA-NC. For tissue analysis, 10 mg of mouse glomeruli were lysed, centrifuged, and transferred to the supernatant for Caspase 9 activity analysis.

### Measurement of calcium (Ca^2+^) flux

The intracellular Ca^2+^ concentration of podocytes were measured by the Fluo-4 AM probe (HY-101896, MedChemExpress). 1 × 10^5^ cells were gathered and stained 1 μM working solution (Ca^2+^-free HBSS) at 37 °C for 1 h protected from light. The cells were then washed thoroughly with Ca²⁺-free HBSS and analyzed using an Attune NxT Acoustic Focusing Cytometer. Data acquisition was followed by analysis with FlowJo software (version 10.4, BD).

### RNA immunoprecipitation (RIP) assay

MPC5 were transfected with vector (pcDNA 3.1) or *Trim7-Flag* plasmid or *Trim7-Flag* truncated mutants for 48 h. Cell extracts produced in lysis buffer (P0013, Beyotime), incubated with antibody of Flag-tag or control IgG for 1 h, then added 50 µL of protein A/G beads at 4 °C for overnight incubation. RIP buffer was used to wash the beads. 20 U RNase-free DNase I and 0.5 mg/mL Proteinase K were used to remove DNA and proteins, respectively. The Trizol was used to purify the RNA, and qRT-PCR was used to normalize the input by calculating the fold change for comparison.

### RNA pull-down

The full-length sequence, 5'untranslated region (UTR), CDS, 3'UTR, as well as 3'UTR mutant of *Bok* was produced and cloned into pUC57-T7 vector designed by GenePharma. PCR products used forward primers that included T7 RNA polymerase promoter sequences. As a DNA template for *in vitro* transcription, purified PCR products were employed. Biotin-labeled *Bok* RNA was generated by transcription utilizing aforementioned DNA template with the T7 promoter following the instructions for a Ribo™ RNAmax-T7 Transcription Kit (C11002-1, RiboBio). Next, 1 μg of purified biotinylated RNA was incubated with 100 μL of streptavidin-conjugated magnetic beads (HY-K0208, MedChemExpress) at room temperature for 30 min. Then, 3 mg protein of MPC5 cell lysates were mixed with cleaned beads, and incubated at room temperature for 1 h. The protein was extracted from beads after washing and boiling in sodium dodecyl sulfate buffer, and then identified by utilizing Western blot analysis.

### ROS assay

As described 25, the DCFH_2_-DA probe (D6883, Sigma-Aldrich) was utilized to detect total ROS levels in MPC5 and HPCs. In a nutshell, 96-well plates MPC5 or HPCs (1 × 10^4^ cells/well) were stained with DCFH_2_-DA at 37 °C for 30 min. The DCFH_2_-DA cell fluorescence intensity was recorded at λex = 488 nm and λem = 525 nm via a microplate reader. To standardize the data of ROS level in MPC5 or HPCs, cell activity was computed.

The mitochondrial ROS (mitoROS) levels were detected using the fluorescence probe MitoSOX (M36008, Thermo). 6-well plates MPC5 or HPCs (2 × 10^5^ cells/well) were gathered and stained 5 μM working solution. MPC5 or HPCs mitoROS levels were evaluated with Focusing Cytometer (Invitrogen) and analyzed via FlowJo 10.4 software (BD).

### Molecular docking

To conduct the molecular docking assay, the structure of protein NLRP6 (6NCV) (Receptor) was obtained from the RSCB Protein Data Bank database (https://www.rcsb.org/). The three-dimensional structure of gastrodin (Ligand) was downloaded from the PubChem database (https://pubchem.ncbi.nlm.nih.gov/). The water molecule was removed from the Receptor, and nonpolar hydrogen was added to Receptor and Ligand by PyMOL (DeLano Scientific LLC) and AutoDockTools (The Scripps Research Institute). AutoDock4 was applied to perform docking process, and the hydrogen bond and charge of Ligand and Receptor were calculated. Visualization of the binding mode was carried out using the Molecular Operating Environment software (Chemical Computing Group ULC).

### Microscale thermophoresis (MST) assay

MST instrument Monolith NT.115 (NanoTemper Technologies, Munich) was used to conduct the binding assay between gastrodin and NLRP6. NLRP6-eGFP cell lysates were ensured that the fluorescence intensity of NLRP6 during the MST assay was about 500RU. Gastrodin (20 μM) was serially diluted 16 times with MST buffer (0.05 % Tween-20). Each dilution was mixed 1:1 with the NLRP6-eGFP lysate and incubated for 10 min at room temperature in the dark. The binding reaction was then loaded into standard glass capillaries, and MST measurement was performed on the NT.115 instrument following the manufacturer's protocol.

### Statistical analysis

The data are presented as the mean ± SEM. The statistical analysis was conducted using GraphPad Prism V.8.0. The two tailed-Student's *t*-test was used to compare two groups. One-way or two-way ANOVA, followed by Tukey's post-test, were used to analyze the differences between multiple groups. The definition of statistical significance was a *P* value of less than 0.05.^ *^P < 0.05,^ **^P < 0.01,^ ***^P < 0.001.

## Results

### Abnormal expression of *Nlrp6/12* is observed in diverse clinical nephropathy samples

**Figure 1:**
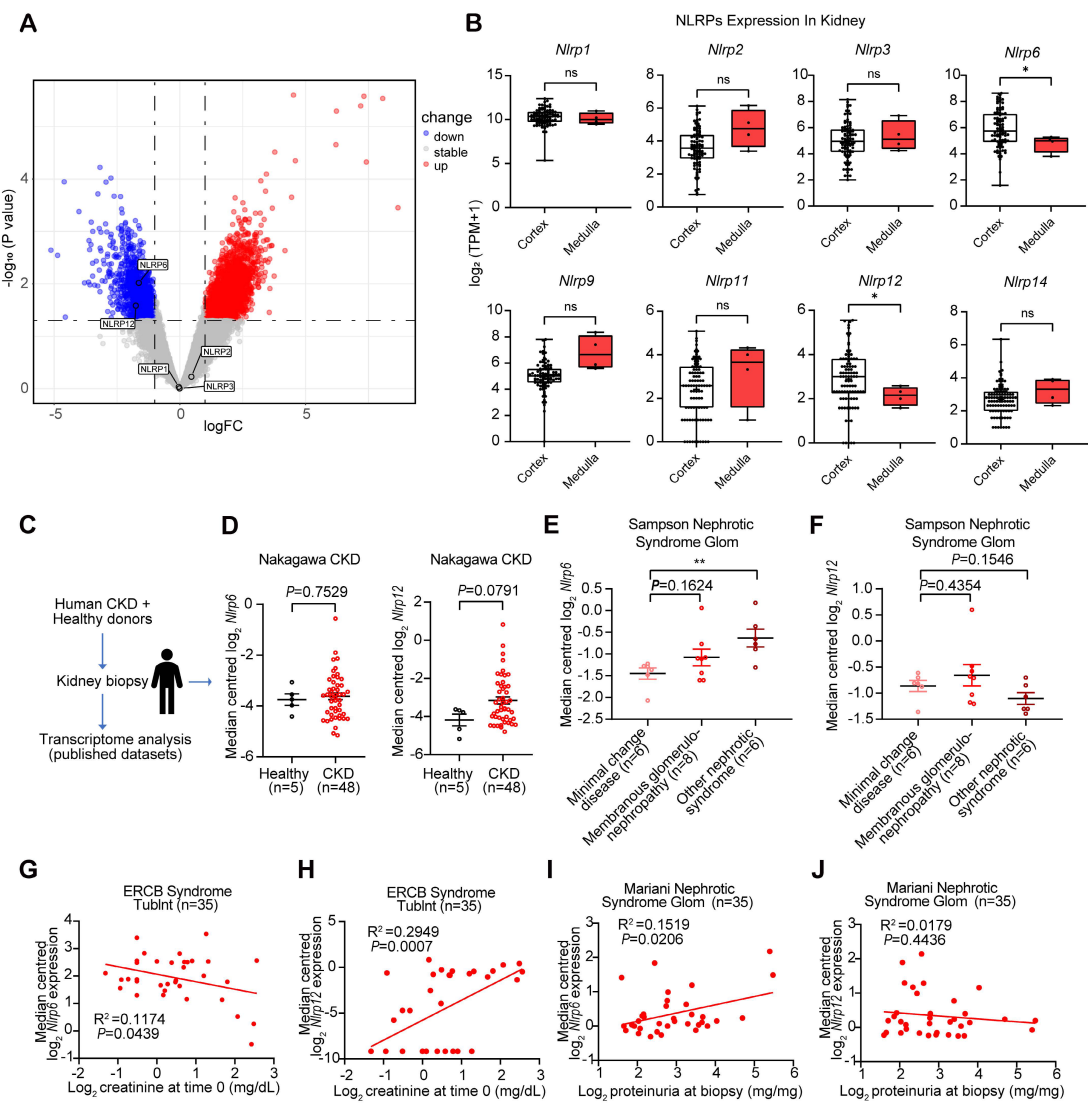
Glomerular *Nlrp6* upregulation was correlated with a decline in renal function. (A) A volcano plot showing the distribution of differential expression genes in the GSE154881 dataset, with NLRPs labeled. (B) Expression of NLRPs in different regions of healthy human kidney tissue (C-D) *Nlrp6* and *Nlrp12* expression in the human renal tissue biopsy of healthy living donors and CKD patients, analyzed by microarray data of Nakagawa CKD kidney dataset. (E-F)* Nlrp6* and *Nlrp12* expression in the human glomeruli of Sampson Nephrotic Syndrome Glom dataset. (G-H) Correlation analysis between* Nlrp6* or* Nlrp12* expression and urine creatinine in European Renal cDNA Bank Nephrotic Syndrome Tublnt dataset, (n = 35). (I-J) Correlation analysis between* Nlrp6* or* Nlrp12* expression and proteinuria in Mariani Nephrotic Syndrome Glom dataset, (n = 35). Data are expressed as the mean ± SEM. ^*^P < 0.05, ^**^P < 0.01.

We performed DEGs analysis using GEO database between diabetic nephropathy (DN) patients (*n* = 5) and healthy volunteers (*n* = 5), and identified 3,572 DEGs including 2,139 upregulated and 1,433 downregulated genes, with *Nlrp6* and *Nlrp12* showing significant downregulation among NLRPs, while *Nlrp1*, *Nlrp2*, and* Nlrp3* exhibited no notable changes. The differential expression genes were visualized in a volcano plot (Fig. 1A). Additionally, we analyzed the expression patterns of NLRPs (including* Nlrp1/2/3/6/9/11/12/14*) in healthy human kidney tissues GTEx database, and found significantly high expression of *Nlrp6* and* Nlrp12* in renal cortex compared with the medulla (Fig. 1B). This consistent spatial expression pattern indicated that *Nlrp6* and *Nlrp12* may have different functions from other NLRPs in the renal cortex.

Next, analysis of transcriptomic data from human kidney biopsies of CKD patients (Nephroseq database) revealed a non-significant trend toward higher expression of *Nlrp6* (*P* = 0.7529) and *Nlrp12* (*P* = 0.0791) expression in CKD patients than that in healthy living donors (Fig. 1C-D). Given the critical role of glomerular structural and functional alteration in CKD progression, we subsequently examined *Nlrp6* and *Nlrp12* expression patterns in glomerular samples from patients with nephrotic syndrome 26. Notably, *Nlrp6* level in membranous glomerulonephropathy was marginally higher than that in MCD, whereas more severe nephrotic syndrome subtypes exhibited statistically significant elevation relative to MCD (*P* < 0.01, Fig. 1E). However, *Nlrp12* expression showed no significant variation across different degrees of glomerular injury (Fig. 1F). Furthermore, linear regression analysis identified a significant inverse correlation between *Nlrp6* expression and urinary creatinine level in nephrotic syndrome patients (*P* = 0.0439, Fig. 1G), along with a positive association with proteinuria (*P* = 0.0206, Fig. 1I). Conversely, *Nlrp12* displayed significant positive correlation with urinary creatinine level (*P* = 0.0007, Fig. 1H) but no significant relationship with proteinuria (*P* = 0.4436, Fig. 1J). These findings suggested a potential association between high *Nlrp6* expression and both glomerular injury severity and renal functional impairment.

### NLRP6 expression is increased in high fructose-induced glomerular injury in mice

**Figure 2:**
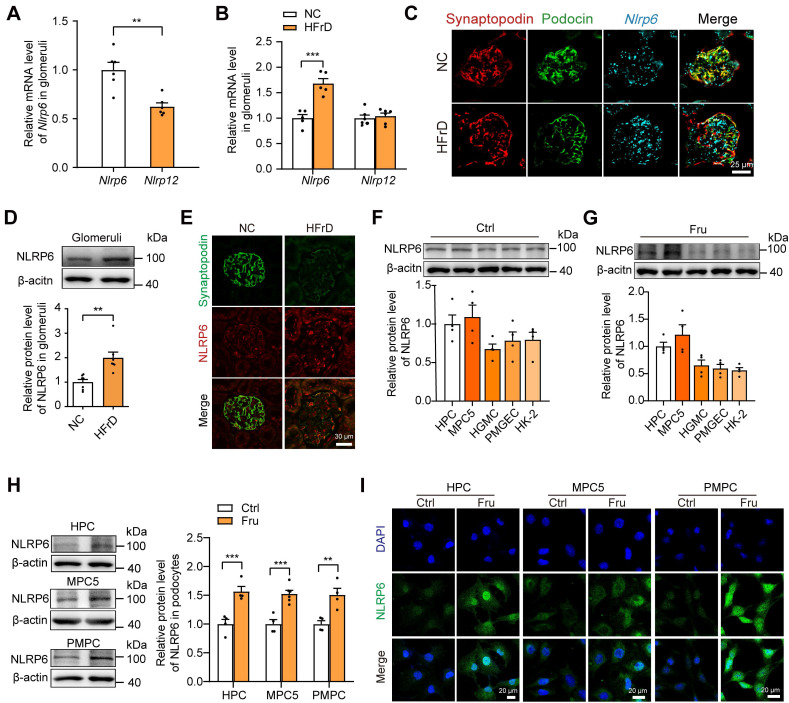
NLRP6 expression is increased in high fructose-induced glomerular podocyte injury in mice (A) *Nlrp*6 and *Nlrp12* mRNA levels in WT mouse glomeruli, (*n* = 6). (B) *Nlrp*6 and *Nlrp12* mRNA levels in WT mouse glomeruli with or without HFrD, (*n* = 4-6). (C) The representative micrographs of RNAscope (*Nlrp6* mRNA, cyan) and IF (Podocin, green; Synaptopodin, red) co-labeled the podocytes of mouse glomeruli. Scale: 25 μm. (D) Western blot detection of NLRP6 in WT mouse glomeruli with or without HFrD, (n = 7). (E) Representative IF images of NLRP6 (green) and Synaptopodin (red) co-labeled the podocytes of mouse glomeruli. Scale: 30 μm. (F) Western blot detection of NLRP6 in kidney cells, (*n* = 4). (G) Western blot detection of NLRP6 in kidney cells exposed to 5 mM fructose, (*n* = 4). (H) Western blot detection of NLRP6 in podocytes (HPCs, MPC5, PMPCs) exposed to 5 mM fructose or not, (*n* = 4-6). (I) Representative IF images of NLRP6 (green) in podocytes (HPCs, MPC5, PMPC) exposed to 5 mM fructose or not. HPCs, Scale: 20 μm; MPC5, Scale: 20 μm; PMPC, Scale: 20 μm. NC: normal chow; HFrD: high fructose diet; HPCs: human podocytes; MPC5: mouse podocyte clone-5 cell; HGMC: human glomerular mesangial cell; PMGEC: primary mouse glomerular endothelial cell; HK-2: human proximal tubule epithelial cell; PMPC: primary mouse podocyte. Data are expressed as the mean ± SEM. ^**^P < 0.01, ^***^P < 0.001.

Subsequently, we detected the variation of glomerular* Nlrp6* and *Nlrp12* expression in healthy mice or high fructose-stimulated mice and we found that *Nlrp6* mRNA level in glomeruli isolated from normal chow mice was much higher than *Nlrp12* (Fig. 2A). Analysis of qRT-PCR, RNAscope *in situ* hybridization, Western blot, and IF staining demonstrated high NLRP6 expression (Fig. 2B-E and Fig. S1A) in glomeruli of mice fed with 12 weeks-HFrD, which were associated with glomerular podocyte injury (Fig. S2A-H). To explore the location of NLRP6 in kidney tissue, we detected its protein expression in different renal cortex cells cultured with or without high fructose, and observed that NLRP6 expression in HPCs and MPC5 was higher than in other cells (HGMCs, PMGECs, and HK-2) (Fig. 2F-G). Thus, we focused on high fructose-stimulated podocytes in subsequent exploration. The data showed that NLRP6 expression was significantly increased in high fructose-cultured HPCs, MPC5, and PMPCs compared with the respective control group (Fig. 2H-I).

### Knockout of *Nlrp6* prevents high fructose-induced glomerular podocyte injury in mice

**Figure 3:**
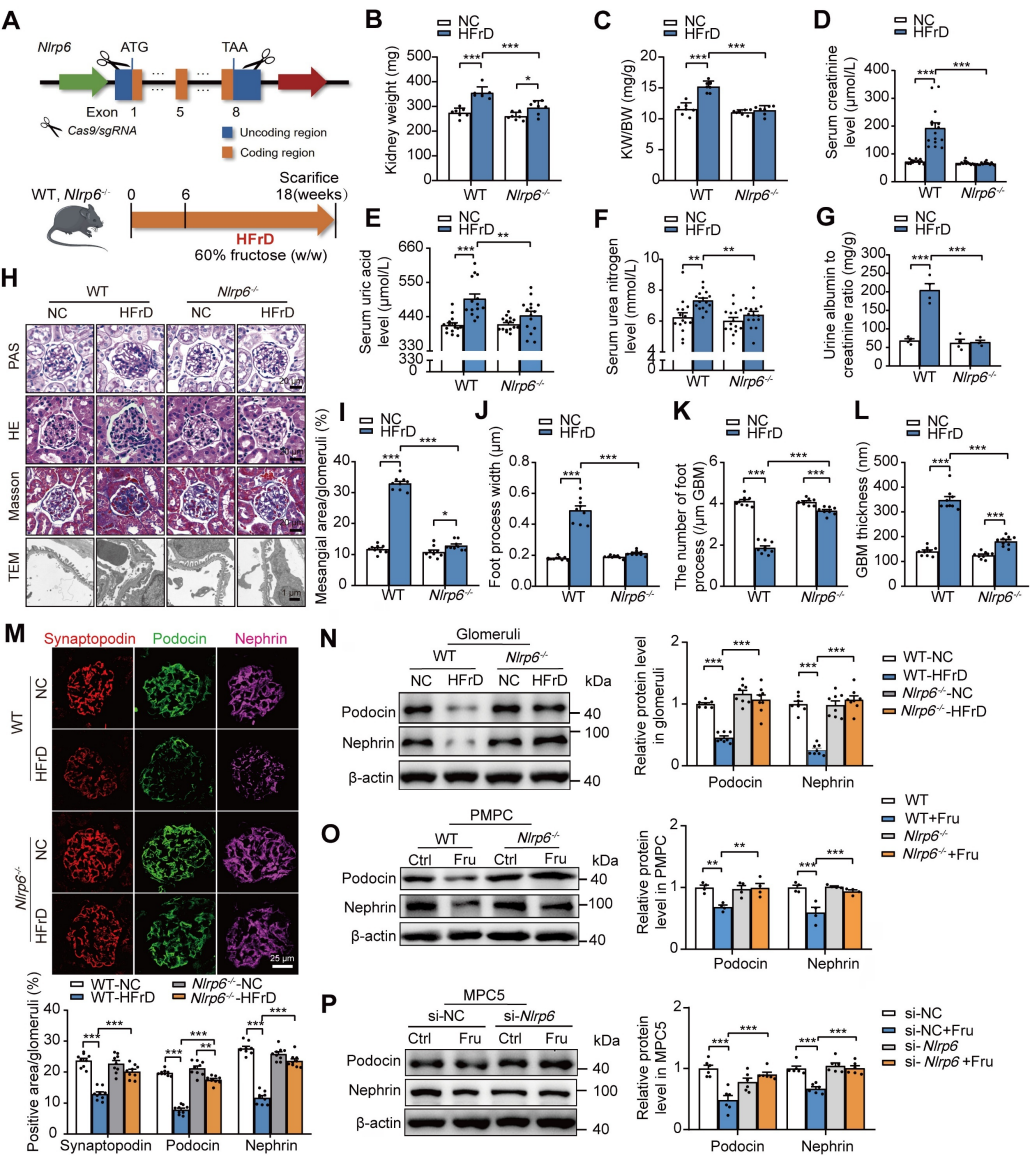
Knockout of *Nlrp6* alleviates high fructose-induced glomerular podocyte injury in mice. (A) A schematic diagram shows the construction of *Nlrp6^-/-^* mouse (top) and a diagram of the procedure of HFrD-fed mouse (bottom). (B-C) The kidney wet weight, and kidney index of WT and *Nlrp6^-/-^* mouse with or without HFrD, (*n* = 7). (D-G) Serum uric acid, creatinine, urea nitrogen, and the ratio of urine albumin to creatinine were measured in WT and *Nlrp6^-/-^* mice with or without HFrD, (*n* = 4-15), respectively. (H-L) Representative images show glomerular changes by morphological examinations, including PAS staining, HE staining, Masson staining, and TEM in WT and *Nlrp6^-/-^* mice with or without HFrD. Scale: PAS, HE, Masson: 20 μm; TEM: 1 μm. (M) Representative images of IF and quantifications of Synaptopodin (red), Podocin(green), and Nephrin (magenta) in WT and *Nlrp6^-/-^* mouse glomeruli with or without HFrD. Scale: 25 μm. (N) Western blot detection of Podocin and Nephrin in WT and *Nlrp6^-/-^* mouse glomeruli with or without HFrD, (*n* = 7-8). (O) Western blot detection of Podocin and Nephrin in PMPCs isolated from WT and *Nlrp6^-/-^* mice, which were stimulated with 5 mM fructose or not, (*n* = 4). (P) Western blot detection of Podocin and Nephrin in MPC5, which were transfected with siRNA-NC or siRNA-*Nlrp6*, subsequently stimulated with 5 mM fructose or not, (*n* = 6). WT: Wild type; *Nlrp6^-/-^*: *Nlr*p6 knockout; KW/BW: kidney weight/body weight; GBM: glomerular basement membrane; Ctrl: Control; Fru: Fructose; si-NC: siRNA-Negative control; si-*Nlrp*6: siRNA-*Nlrp*6. Data are expressed as the mean ± SEM. ^*^P < 0.05, ^**^P < 0.01, ^***^P < 0.001.

To explore the potential impact of NLRP6 on glomerular podocyte injury, we successfully produced *Nlrp6^-/-^* mice by CRISPR/Cas9 technology (Fig. 3A). The efficiency of *Nlrp6^-/-^* was further validated by IF, qRT-PCR, and Western blot assays of isolated mouse glomeruli (Fig. S3A-C). There were no significant differences in kidney physiological structure and metabolic function between* Nlrp6^-/-^* mice and WT mice (Fig. S3D-K)*.* Then, we fed HFrD to WT and *Nlrp6^-/-^* mice for 12 weeks, and control group with normal chow, and then assessed the severity of glomerular podocyte injury (Fig. 3A). HFrD-fed *Nlrp6^-/-^* mice showed lower kidney weight, serum level of uric acid, creatinine, urea nitrogen level, and urinary albumin excretion (Fig. 3B-G) as well as slighter glomerular podocyte injury (Fig. 3H-L) than that in HFrD-fed WT mice. Decreased expression of podocyte-structural proteins Nephrin, Podocin, and Synaptopodin in glomeruli induced by HFrD feeding was rescued in *Nlrp6^-/-^* mice compared with WT mice (Fig. 3M-N). We isolated PMPCs from mouse glomeruli of WT and *Nlrp6^-/-^* mice and exposed them to high fructose, respectively, and found that *Nlrp6^-/-^* reversed high fructose-caused downregulation of Podocin and Nephrin (Fig. 3O). Confirmedly, MPC5 transfected with siRNA-*Nlrp6* attenuated high fructose-caused downregulation of Podocin and Nephrin expression, compared with siRNA-NC group (Fig. 3P). Together, these data indicated that the absence of *Nlrp6* ameliorated high fructose-induced glomerular podocyte injury.

### NLRP6 inhibition attenuates podocyte mitochondria-mediated apoptosis under high fructose stimulation

**Figure 4:**
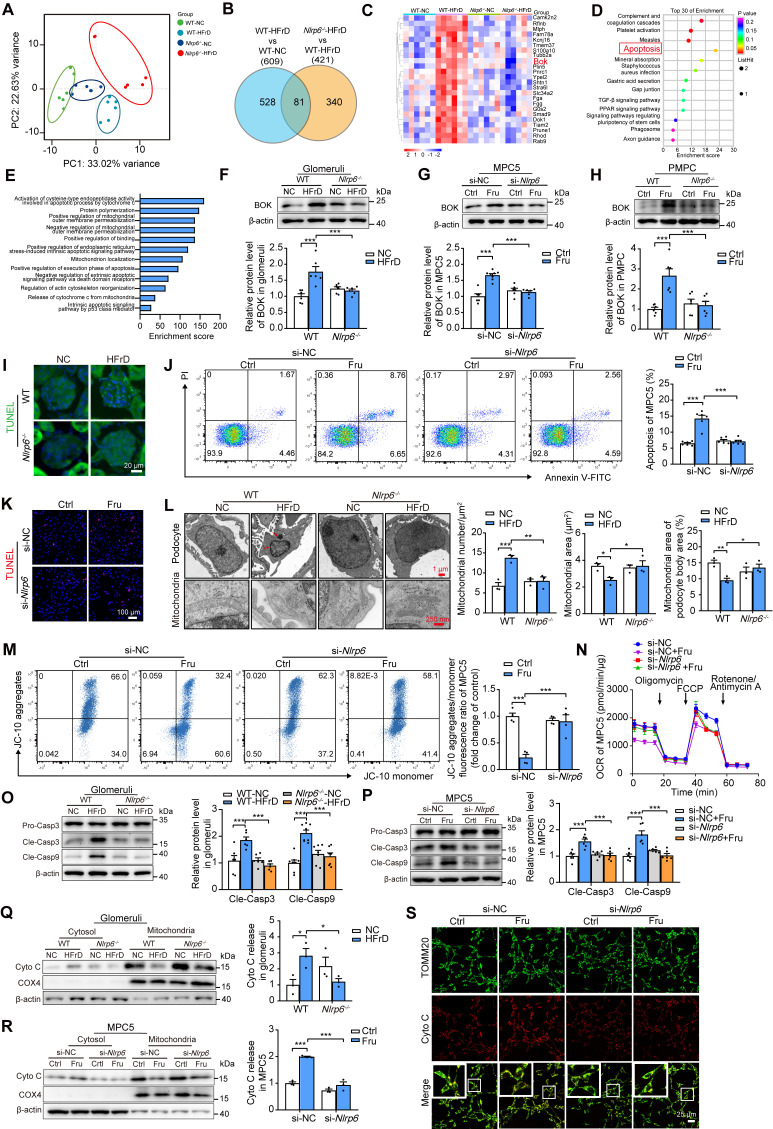
NLRP6 inhibition attenuates mitochondria-mediated apoptosis under high fructose stimulation. (A) The principal component analysis of WT and *Nlrp6^-/-^* mouse glomeruli with or without HFrD, (*n* = 6). (B) Venn diagram shows the overlapped unique differentially expressed genes between WT-HFrD versus WT-NC and *Nlrp6^-/-^*-HFrD versus WT-HFrD from the data of RNA-seq analysis of mouse-isolated glomeruli. (C-E) Heatmap shows the overlapped gene (downregulation), and analyzed by KEGG and GO. (F) Western blot detection of BOK in WT and *Nlrp6^-/-^* mouse glomeruli with or without HFrD, (*n* = 6). (G) Western blot detection of BOK in MPC5, which was stimulated with or without 5 mM fructose after being transfected with siRNA-NC or siRNA-*Nlrp6*, (*n* = 6-8). (H) Western blot detection of BOK in PMPCs isolated from WT and *Nlrp6^-/-^* mice, which were stimulated with or without 5 mM fructose, (*n* = 6). (I) Representative IF images of TUNEL assay in WT and *Nlrp6^-/-^* mouse glomeruli with or without HFrD, Scale: 20 μm. (J) Flow cytometry analysis of apoptotic cells through Annexin V-FITC/PI staining. MPC5 were stimulated with or without 5 mM fructose after being transfected with siRNA-NC or siRNA-*Nlrp6*, (*n* = 6). (K) Representative IF images of TUNEL assay in MPC5, which was stimulated with or without 5 mM fructose after being transfected with siRNA-NC or siRNA-*Nlrp6*, Scale: 100 μm. (L) TEM images and quantification of podocyte apoptotic morphology and mitochondria in WT and *Nlrp6^-/-^* mouse glomeruli with or without HFrD. Scale: 1 μm (Top), 250 nm (Bottom), (*n* = 3). (M) Flow cytometry analysis of mitochondrial membrane potentials using JC-10 dye. MPC5 were stimulated with or without 5 mM fructose after being transfected with siRNA-NC or siRNA-*Nlrp6*, (*n* = 4). (N) OCR detection in MPC5, which was stimulated with or without 5 mM fructose after being transfected with siRNA-NC or siRNA-*Nlrp6*, (*n* = 4-6). (O)Western blot detection of cleaved Caspase 3 and cleaved Caspase 9 in WT and *Nlrp6^-/-^* mouse glomeruli with or without HFrD, (*n* = 6-8). (P) Western blot detection of cleaved Caspase 3 and cleaved Caspase 9 in MPC5, which were stimulated with or without 5 mM fructose after being transfected with siRNA-NC or siRNA-*Nlrp6*, (*n* = 6). (Q) Western blot detection of Cyto C performed on mitochondrial and cytosolic fractions in WT and *Nlrp6^-/-^* mouse glomeruli with or without HFrD, (*n* = 3). (R) Western blot detection of Cyto C was performed on mitochondrial and cytosolic fractions in MPC5, which were stimulated with or without 5 mM fructose after being transfected with siRNA-NC or siRNA-*Nlrp6*, (*n* = 3). (S) IF analysis of Cyto C and TOMM20 in MPC5, which were stimulated with or without 5 mM fructose after being transfected with siRNA-NC or siRNA-*Nlrp6*. Scale: 25 μm. CASP: Caspase; PI: propidium iodide; TOMM20, translocase of the outer membrane 20; Cyto C: Cytochrome C. Data are expressed as the mean ± SEM. ^*^P < 0.05, ^**^P < 0.01, ^***^P < 0.001.

To investigate the molecular mechanism of how *Nlrp6* deficiency attenuates podocyte injury, we performed RNA-seq to identify differentially expressed genes of glomeruli between HFrD-fed WT mice versus normal chow-fed WT mice, and HFrD-fed *Nlrp6^-/-^* mice versus HFrD-fed WT mice. The principal component analysis showed that different expression spectrums of glomeruli were well distinguished in these four groups (Fig. 4A). 609 genes were differentially expressed in glomeruli of HFrD-fed WT mice versus normal chow-fed WT mice, and 421 genes were differentially expressed in glomeruli of HFrD-fed *Nlrp6^-/-^* mice versus HFrD-fed WT mice (Fig. 4B). After intersecting the above two sets of differentially expressed genes, we identified 81 overlapping genes and selected the following genes: upregulated under high fructose stimulation and downregulated after *Nlrp6* knockout. The downregulated genes were shown in heatmap (Fig. 4C), followed by analysis of Kyoto Encyclopedia of Genes and Genomes (KEGG) and Gene Ontology (GO) (Fig. 4D-E), respectively. KEGG analysis indicated that the downregulation of apoptosis pathway had the highest enrichment score in HFrD-fed *Nlrp6^-/-^* mice compared with HFrD-fed WT mice (Fig. 4D). GO analysis exhibited that apoptosis-related biological process, including positive regulation of Cyto C release from mitochondria. This positive regulation of apoptotic process was notably inhibited in HFrD-fed *Nlrp6^-/-^* mice compared with HFrD-fed WT mice (Fig. 4E).

This led us to hypothesize that NLRP6 inhibition may decrease glomerular podocyte apoptosis under high fructose stimulation. The heatmap showed significant downregulation of the* Bok* gene in HFrD-fed *Nlrp6^-/-^* mice compared with HFrD-fed WT mice (Fig. 4C), indicating that BOK may be a crucial checkpoint in NLRP6-related podocyte apoptosis through mitochondria-dependent pathway. In fact, high fructose stimulation increased BOK expression in mouse glomeruli, MPC5 and PMPCs (Fig. 4F-H). *Nlrp6* deficiency blocked high fructose-induced upregulation of BOK *in vivo* and *in vitro* (Fig. S4A-B).

We conducted further investigation to determine if *Nlrp6* deficiency could protect against high fructose-induced podocyte mitochondria-mediated apoptosis. Indeed, *Nlrp6* deficiency strongly suppressed high fructose-induced glomerular podocyte apoptosis measured by TUNEL assay in MPC5 and glomeruli, and fluorescein isothiocyanate-conjugated Annexin V-FITC/PI staining in MPC5 and PMPCs (Fig. 4I-K and Fig. S5A), respectively. *Nlrp6* deficiency prevented glomerular podocyte apoptotic features, including pronounced cell shrinkage, margination and condensation of chromatin at the nuclear periphery, and mitochondrial structure destruction (presenting mitochondrial fragmentation, swelling deformation, and cristae loss) in HFrD-fed mice (Fig. 4L). One of the hallmarks of early apoptotic cells is the irreversible decrease of mitochondrial membrane potential (ΔΨm). Given that MOMP is related to the ΔΨm, we showed that siRNA-*Nlrp6* or *Nlrp6* knockout reversed high fructose-caused ΔΨm reduction in podocytes compared with control group under high fructose stimulation (Fig. 4M and Fig. S5B). OCR decrease is a direct quantification of mitochondrial respiration injury. We observed that *Nlrp6* deficiency ameliorated high fructose-induced decrease of OCR in MPC5 (Fig. 4N). Insufficient or excessive Ca^2+^ fluxes cause cell apoptosis. We used the Fluo-4 AM probe to detect the intracellular Ca^2+^ concentration of MPC5 in the presence of NLRP6 interference. Compared with the siRNA-NC group, high fructose induced a slight increase of Ca^2+^ concentration in podocytes. Importantly, when *Nlrp6* was knocked down, there was no significant difference in Ca^2+^ concentration compared with siRNA-NC group, suggesting that NLRP6 expression change alone did not alter baseline Ca^2+^ concentration. Furthermore, no significant decrease of intracellular Ca^2+^ concentration was observed upon *Nlrp6* knockdown compared with siRNA-NC group under high fructose exposure (Fig. S6A), indicating that NLRP6 rarely interfered with intracellular Ca^2+^ concentration in podocytes with or without high fructose stimulation. MOMP triggers the release of Cyto C into the cytosol and subsequent Caspase activation 7. Compared with HFrD-fed WT mice or high fructose-cultured podocytes, the absence of *Nlrp6* significantly attenuated high fructose-induced cleavage of Caspase 3 and Caspase 9 (Fig. 4O-P and Fig. S5C), as well as high activity of Caspase 3 and Caspase 9 in mouse glomeruli and MPC5 (Fig. S6B-D). We also found an increase of Cyto C release in high fructose-stimulated mouse glomeruli and PMPCs compared with control group, while *Nlrp6* knockout significantly blocked high fructose-induced release of Cyto C in mouse glomeruli and PMPCs (Fig. 4Q and Fig. S5D). Colocalization analysis and Western blot confirmed that high fructose-induced Cyto C leakage from MPC5 mitochondria was alleviated by siRNA-*Nlrp6*, compared with siRNA-NC group (Fig. 4R-S). These findings, along with the results mentioned, suggested that NLRP6 downregulation restrained high fructose-induced podocyte mitochondria-mediated apoptosis.

### NLRP6 inhibition upregulates TRIM7 to decrease *Bok* mRNA stability in podocytes

**Figure 5:**
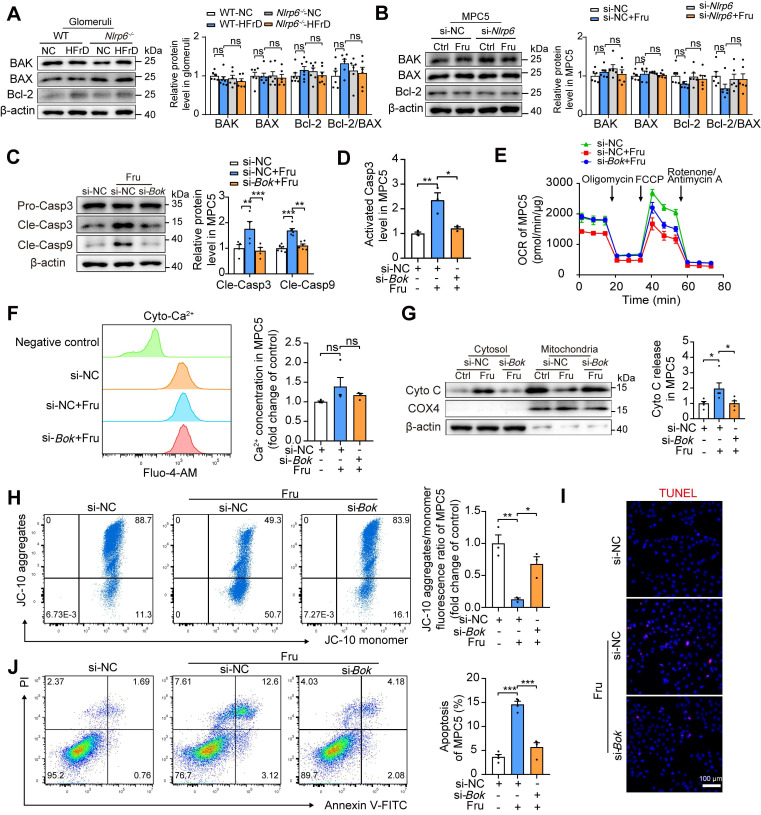
NLRP6 inhibition relieves mitochondria-mediated apoptosis by downregulating BOK in high fructose-stimulated podocytes. (A) Western blot detection of BAK, BAX and Bcl-2 in WT and *Nlrp6^-/^*^-^ mouse glomeruli with or without HFrD, (*n* = 6-7). (B) Western blot detection of BAK, BAX and Bcl-2 in MPC5, which were stimulated with or without 5 mM fructose after being transfected with siRNA-NC or siRNA-*Nlrp6*, (*n* = 6). (C) Western blot detection of cleaved Caspase 3 and cleaved Caspase 9 in MPC5, which were stimulated with or without 5 mM fructose after being transfected with siRNA-NC or siRNA-*Bok*, (*n* = 4-6). (D) Flow cytometry analysis of activated Caspase 3 in MPC5, which were stimulated with or without 5 mM fructose after being transfected with siRNA-NC or siRNA-*Bok*, (*n* = 3). (E) OCR detection in MPC5, which was stimulated with or without 5 mM fructose after being transfected with siRNA-NC or siRNA-*Bok*, (*n* = 6). (F) Flow cytometry analysis of total intracellular Ca^2+^ concentration using Fluo-4 AM (1 μM) in MPC5, which were stimulated with or without 5 mM fructose after being transfected with siRNA-NC or siRNA-*Bok*, (*n* = 4). (G) Western blot detection of Cyto C was performed on mitochondrial or cytosolic fractions in MPC5, which were stimulated with or without 5 mM fructose after being transfected with siRNA-NC or siRNA-*Bok*, (*n* = 5). (H) Flow cytometry analysis of mitochondrial membrane potentials using JC-10 dye in MPC5, which were stimulated with or without 5 mM fructose after being transfected with siRNA-NC or siRNA-*Bok*, (*n* = 3). (I) Representative IF images of TUNEL assay in MPC5, which were stimulated with or without 5 mM fructose after being transfected with siRNA-NC or siRNA-*Bok*, Scale: 100 μm. (J) Flow cytometry analysis of apoptotic cells through Annexin V-FITC/PI staining. MPC5 were stimulated with or without 5 mM fructose after being transfected with siRNA-NC or siRNA-*Bok*, (*n* = 4). Data are expressed as the mean ± SEM. ^*^P < 0.05, ^**^P < 0.01, ^***^P < 0.001. no significance, ns.

BOK dominates cell apoptosis via mitochondria-mediated pathway. To verify if the protective action of *Nlrp6* deficiency was dependent on BOK downregulation, not main pro-apoptotic effector of Bcl-2 family or anti-apoptotic effector Bcl-2, we detected and found that *Nlrp6* deficiency did not alter BAK, BAX and Bcl-2 protein expression, as well as Bcl-2/BAX ratio in HFrD-fed mouse glomeruli and high fructose-cultured podocytes (Fig. 5A-B). To explore whether BOK downregulation mitigated podocyte MOMP, siRNA-NC or siRNA-*Bok* was transfected into MPC5. The inhibition of BOK dramatically decreased high levels of Caspase 3 and Caspase 9 cleavage, as well as activated Caspase 3, compared with siRNA-NC group in MPC5 stimulated with high fructose (Fig. 5C-D). The transfection of siRNA-*Bok* rescued high fructose-induced podocyte mitochondrial respiration impairment (Fig. 5E), but had no effect on Ca^2+^ flux regulation (Fig. 5F). Moreover, high fructose-induced excessive release of Cyto C, mitochondrial membrane potential abnormality, and apoptosis were inhibited by siRNA-*Bok*, resulting in a lower abundance of Cyto C in the cytoplasm, higher level of membrane potential, and less apoptosis than siRNA-NC group in podocytes (Fig. 5G-J). Additionally, we assessed apoptosis levels in podocytes subjected to *Bok* silencing based on NLRP6 overexpression.

In comparison to NLRP6-overexpressed group, the silencing of *Bok* effectively attenuated the upregulation of cleaved Caspase 3, cleaved Caspase 9, activated Caspase 3, and the leakage of Cyto C induced by NLRP6 overexpression in MPC5 (Fig. S7A-B, D). BOK inhibition attenuated NLRP6 overexpression-induced the decrease of ΔΨm and the increase of apoptosis in MPC5 (Fig. S7C, E-F). Of note, PINK1-Parkin axis-mediated mitophagy activation protects against podocyte apoptosis induced by palmitic acid, puromycin aminonucleoside, and cadmium 27-29. Therefore, we explored in-depth whether* Nlrp6* deficiency was sufficient to exert an anti-apoptosis effect by increasing mitophagy. As shown in Fig. S8A and B, compared with control group, high fructose did not significantly alter PINK1 and Parkin expression, and *Nlrp6* deficiency also did not affect PINK1 and Parkin expression in mouse glomeruli and MPC5. BOK engages mitophagy to alleviate mitochondrial malfunction in hippocampal neurons of mice with Alzheimer's disease 30. However, our study showed that knockdown of *Bok* did not regulate PINK1 and Parkin expression in podocytes exposed to high fructose (Fig. S8C). These results suggested that *Nlrp6* deficiency mainly suppressed BOK to reduce high fructose-induced podocyte mitochondria-mediated apoptosis, possibly rather than through enhancing PINK1/Parkin-mediated mitophagy.

**Figure 6:**
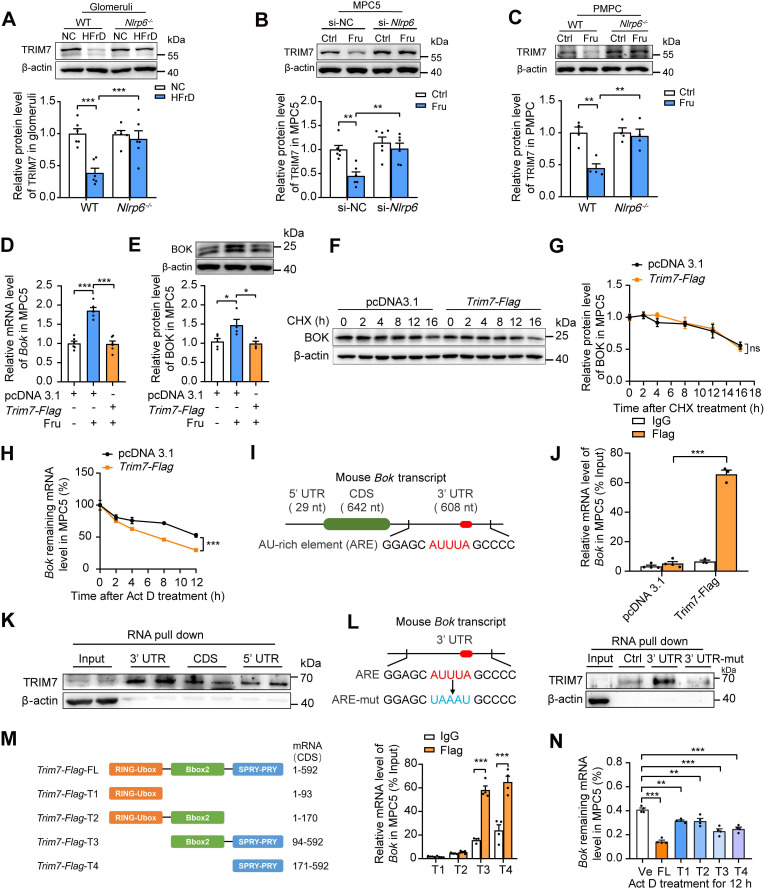
NLRP6 inhibition upregulates TRIM7 to decrease *Bok* mRNA stability in podocytes. (A) Western blot detection of TRIM7 expression in WT and *Nlrp6^-/-^* mouse glomeruli with or without HFrD, (*n*=6). (B) Western blot detection of TRIM7 in MPC5, which was stimulated with or without 5 mM fructose after being transfected with siRNA-NC or siRNA-*Nlrp6*, (*n* = 6). (C) Western blot detection of TRIM7 in PMPCs isolated from WT and *Nlrp6^-/-^* mice, which were stimulated with or without 5 mM fructose after being transfected with siRNA-NC or siRNA-*Nlrp6*, (*n* = 6). (D) *Bok* mRNA level in MPC5, which was stimulated with or without 5 mM fructose after being transfected with vector or *Trim7-Flag*, (*n* = 6). (E) Western blot detection of BOK in MPC5, which were stimulated with or without 5 mM fructose after being transfected with vector or *Trim7-Flag*, (*n* = 4-5). (F-G) Western blot detection of BOK in MPC5 exposed to 5 μg/mL CHX or not for different times after being transfected with vector or *Trim7-Flag*, (*n* = 3). (H)* Bok* remaining mRNA level in MPC5 exposed to 5 μg/mL Act D or not for different times after being transfected with vector or *Trim7-Flag*, (*n* = 6-9). (I) The schematic diagram shows mouse *Bok* mRNA with AU-rich element (ARE). (J) RIP to assess the connection between *Bok* mRNA and TRIM7. Cell lysates from MPC5 transfected with vector or *Trim7-Flag* were immunoprecipitated with IgG or Flag-tag, The mRNA level of *Bok* recruited to TRIM7 was examined by qRT-PCR, (*n* = 3-4). (K) RNA pull-down subsequently followed by Western blot. Biotin-labeled 3'UTR, CDS, and 5'UTR of *Bok* mRNA were incubated with MPC5 cell lysates. Following pull-down, the recruitment of TRIM7 to *Bok* mRNA was examined by Western blot. (L) Left, the schematic diagram shows the mutant sites of mouse *Bok* mRNA ARE region. Right, RNA pull-down was subsequently followed by Western blot. Biotin-labeled 3'UTR, 3'UTR mutant of *Bok* mRNA or Negative control transcript were incubated with MPC5 cell lysates. Following pull-down, the recruitment of TRIM7 to *Bok* mRNA was examined by Western blot. (M) Left, the schematic diagram shows different truncated mutants of mouse *Trim7-Flag* plasmid*.* Right, RIP to assess the connection between *Bok* mRNA and TRIM7 truncated mutants. Cell lysates from MPC5 transfected with *Trim7-Flag* truncated mutants were immunoprecipitated with IgG or Flag-tag. The mRNA level of *Bok* recruited to Trim7 truncated mutants was examined by qRT-PCR, (*n* = 3-4). (N) Remaining *Bok* mRNA level in MPC5, which were transfected with vector or *Trim7-Flag* Full length or* Trim7-Flag* truncated mutants, and subsequently cultured with 5 μg/mL Act D after 12 h, (*n* = 4). Vec: Vector; FL: Full length; T1: Truncated mutant 1; T2: Truncated mutant 2; T3: Truncated mutant 3; T4: Truncated mutant 4. Data are expressed as the mean ± SEM. ^*^P < 0.05, ^**^P < 0.01, ^***^P < 0.001. no significance, ns.

Research on the molecular mechanisms that regulate BOK abundance is limited. TRIM7, an E3 ubiquitin ligase, predominantly ubiquitinates and degrades proteins 31. Interestingly, downregulation of TRIM7 (at both protein and mRNA levels) was observed in HFrD-fed mouse glomeruli and high fructose-cultured MPC5 and PMPCs, respectively, while the absence of *Nlrp6* rescued high fructose-caused TRIM7 low-expression (Fig. 6A-C and Fig. S9A-C) in glomeruli and podocytes. Moreover, protein decay and RNA decay experiments showed that NLRP6 mainly suppressed TRIM7 by prompting *Trim7* mRNA degradation, not by post-translational modification of proteins (Fig. S9D-E). Therefore, we further explored whether NLRP6 inhibition-decreased BOK was regulated by TRIM7. Podocytes were transfected with empty plasmid or *Trim7-Flag* and found that TRIM7 overexpression dramatically decreased BOK expression in high fructose-exposed MPC5, compared with empty plasmid (Fig. 6D-E). Next, to find out if the suppressive impact of TRIM7 on BOK was at the transcript or protein level, a protein synthesis inhibitor CHX was employed to determine whether BOK reduction was caused by proteasomal degradation. There was no significant change in BOK protein degradation in TRIM7-overexpressed MPC5 compared with the empty plasmid (Fig. 6F-G). To limit mRNA transcription, we exposed MPC5 to Act D. mRNA decay assay showed that *Trim7-Flag* raised the *Bok* mRNA decay rate compared with the empty plasmid (Fig. 6H). The 3'UTR of mouse *Bok* mRNA is reported to have a cluster (GGAGCAUUUAGCCCC) of the ARE motif (Fig. 6I) 32, which may serve as a potential binding target for TRIM7 in podocytes. Hence, we speculated that TRIM7 might function as an RNA-binding protein that regulated *Bok* mRNA level in podocytes. RIP-qPCR analysis showed that *Bok* mRNA was enhanced in anti-TRIM7 immunoprecipitates, compared with IgG (Fig. 6J), and TRIM7 protein mostly attached to *Bok* in mRNA 3'UTR, not the CDS or 5'UTR region, based on the point mutation strategy of 3'UTR (a mutation of AUUUA to UAAAU) and RNA pull-down assay (Fig. 6K-L). To explore the specific binding sites of TRIM7 with *Bok* mRNA in podocytes, truncated mutants of Flag-tagged TRIM7 were fabricated (T1-T4), and RIP was performed to verify the effect of the four truncated regions on the stability of *Bok* mRNA in MPC5. *Bok* mRNAs were largely concentrated in TRIM7 mutants containing the PRY/SPRY domains (T3 and T4), which promoted the decay of *Bok* mRNA in MPC5 (Fig. 6M-N). Overall, these results indicated that NLRP6 inhibition downregulated BOK by facilitating *Bok*'s conserved ARE-containing 3'UTR interaction with TRIM7, attenuating high fructose-induced podocyte mitochondria-mediated apoptosis.

### NLRP6 downregulation reduces podocyte mitochondrial ROS and apoptosis via increasing FAM213A antioxidant activity

The excessive production of ROS sensitizes cells to MOMP, disrupting Cyto C release coordination and aggravating mitochondria-mediated apoptosis 33. To investigate the connection between BOK-induced MOMP and ROS production, MPC5 were transfected with empty plasmid or *Bok-HA*, and observed the increase of total and mitochondrial ROS, and decrease of OCR in MPC5 with BOK overexpression in comparison with empty plasmid transfected group (Fig. S10A-C). Hence, we used mitochondria-targeted antioxidant agent MitoTEMPO to block mitoROS generation in podocytes. The inhibition of mitoROS accumulation by MitoTEMPO relieved the drop of ΔΨm in MPC5 transfected with *Bok-HA* plasmid (Fig. S10D). Then, blocking ROS generation by MitoTEMPO ameliorated Caspase 3 activation and apoptosis induced by *Bok-HA* plasmid in podocytes (Fig. S10E-G). These observations indicated that reducing mitoROS production might decelerate BOK-induced podocyte mitochondria-mediated apoptosis.

**Figure 7:**
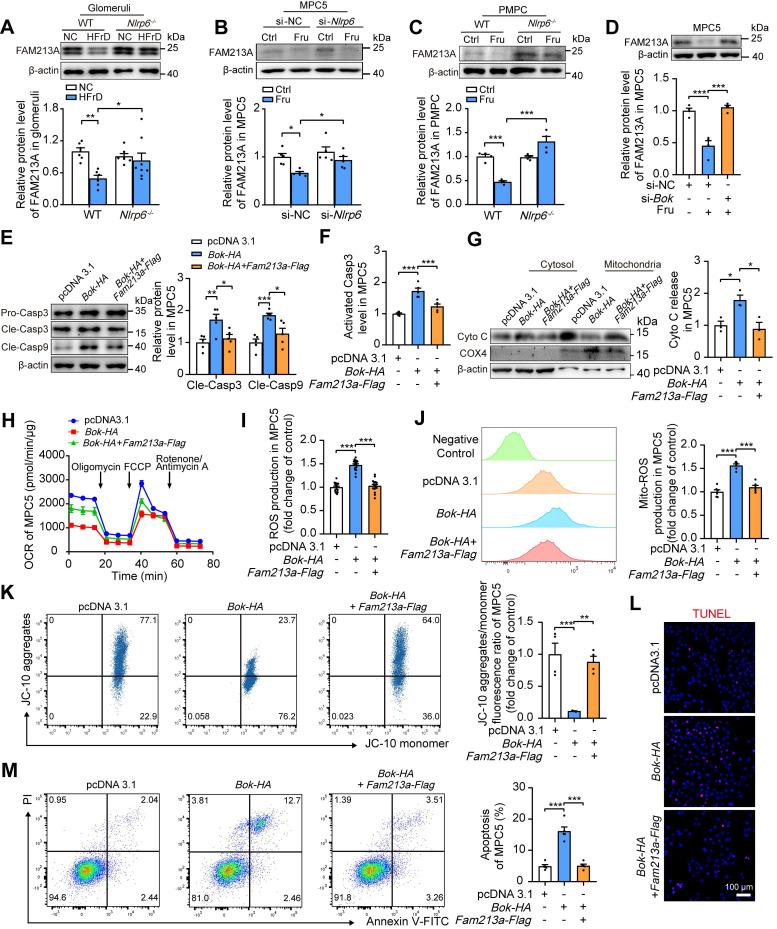
NLRP6 downregulation reduces podocyte mitochondrial ROS and apoptosis via improving FAM213A antioxidant activity. (A) Western blot detection of FAM213A in WT and *Nlrp6^-/-^* mouse glomeruli with or without HFrD, (*n* = 6-8). (B) Western blot detection of FAM213A in MPC5, which was stimulated with or without 5 mM fructose after being transfected with siRNA-NC or siRNA-*Nlrp6*, (*n* = 5). (C) Western blot detection of FAM213A in PMPCs isolated from WT and *Nlrp6^-/-^* mice, which were stimulated with or without 5 mM fructose, (*n* = 4). (D) Western blot detection of FAM213A in MPC5, which were transfected with stimulated with or without 5 mM fructose after being transfected with siRNA-NC or siRNA-*Bok*, (*n* = 4). (E) Western blot detection of cleaved Caspase 3 and cleaved Caspase 9 in MPC5 transfected with vector or *Bok-HA* or *Fam213a-Flag*, (*n* = 5). (F) Flow cytometry analysis of activated Caspase 3 in MPC5 transfected with vector or *Bok-HA* or *Fam213a-Flag*, (*n* = 5). (G) Western blot detection of Cyto C performed on mitochondrial and cytosolic fractions in MPC5 transfected with vector or *Bok-HA* or *Fam213a-Flag*, (*n* = 3). (H) OCR detection in MPC5 transfected with vector or *Bok-HA* or *Fam213a-Flag*, (*n* = 4). (I) ROS levels were considered as the fluorescence intensity of fluorogenic probe DCFH_2_-DA, measured via microplate reader in MPC5 transfected with vector or *Bok-HA* or *Fam213a-Flag*, (*n* = 18). (J) Mitochondrial ROS production was considered as the fluorescence intensity of labeling fluorogenic probe MitoSOX, measured by flow cytometry in MPC5 transfected with vector or *Bok-HA* or *Fam213a-Flag*, (*n* = 6). (K) Flow cytometry analysis of mitochondrial membrane potentials using JC-10 dye in MPC5 transfected with vector or *Bok-HA* or *Fam213a-Flag*, (*n* = 4). (L) Representative IF images of TUNEL assay in MPC5 transfected with vector or *Bok-HA* or *Fam213a-Flag*, Scale: 100 μm. (M) Flow cytometry analysis of apoptotic cells through Annexin V-FITC/PI staining in MPC5 transfected with vector or *Bok-HA* or *Fam213a-Flag*, (*n* = 5). Data are expressed as the mean ± SEM. ^*^P < 0.05, ^**^P < 0.01, ^***^P < 0.001.

Our previous study identified a single antioxidant gene, FAM213A 5, that was consistently downregulated in glomeruli of HFrD-fed rats versus normal controls via Proteomic and RNA-seq analysis (Fig. S11A). Further examination showed that FAM213A was predominantly expressed in mitochondria of mouse glomeruli and MPC5 (Fig. S11B-C). Thus, we hypothesized that the downregulation of FAM213A may contribute to high fructose-induced ROS accumulation. Indeed, high fructose decreased FAM213A expression in mouse glomeruli and podocytes, which was prevented by the absence of *Nlrp6* (Fig. 7A-C). Notably, siRNA-*Bok*, but not siRNA-NC, increased FAM213A protein level in high fructose-cultured MPC5 (Fig. 7D), while *Fam213a-Flag* showed no disturbance on BOK expression (Fig. S12A-B), suggesting that BOK negatively regulated FAM213A expression in podocytes. To further explore whether FAM213A overexpression could reverse BOK-induced mitochondria-mediated apoptosis, we transfected both the *Fam213a-Flag* plasmid and the *Bok-HA* plasmid into MPC5. Overexpressed FAM213A suppressed excessive activation of Caspase 3 and Caspase 9, Cyto C leakage, and OCR decrease caused by *Bok-HA* in MPC5 (Fig. 7E-H). High expression of FAM213A reduced total or mitochondrial ROS overproduction induced by *Bok-HA* in MPC5 (Fig. 7I-J). Moreover, the overexpression of FAM213A mitigated *Bok-HA*-induced podocyte ΔΨm decrease and apoptosis increase (Fig. 7K-M). These findings suggested that BOK inhibition reduced mitoROS accumulation by upregulating FAM213A expression, protecting *Nlrp6*-overexpressing podocytes against high fructose-induced apoptosis.

### Inhibition of NLRP6 by gastrodin ameliorates high fructose-induced glomerular podocyte mitochondria-mediated apoptosis

Here, we evaluated whether the pharmacologically relevant compound prevented mitochondria-mediated apoptosis in glomerular podocytes. Gastrodin is reported to maintain cellular mitochondrial structure and function by reducing oxidative stress 13, 15. We found that gastrodin strongly reduced kidney weight, serum levels of uric acid, creatinine, and urea nitrogen, as well as lowered the ratio of urine albumin to creatinine (Fig. S13A-G) in mice fed with HFrD compared with control group. It also alleviated glomerular podocyte injury like foot process effacement, along with kidney histopathological morphologic alteration in this animal model (Fig. S13H-L). Gastrodin markedly up-regulated Nephrin, Podocin, and Synaptopodin protein levels in mouse glomeruli, MPC5, and HPCs under high fructose exposure (Fig. S13M-P). These data indicated that gastrodin attenuated high fructose-induced glomerular podocyte injury.

**Figure 8:**
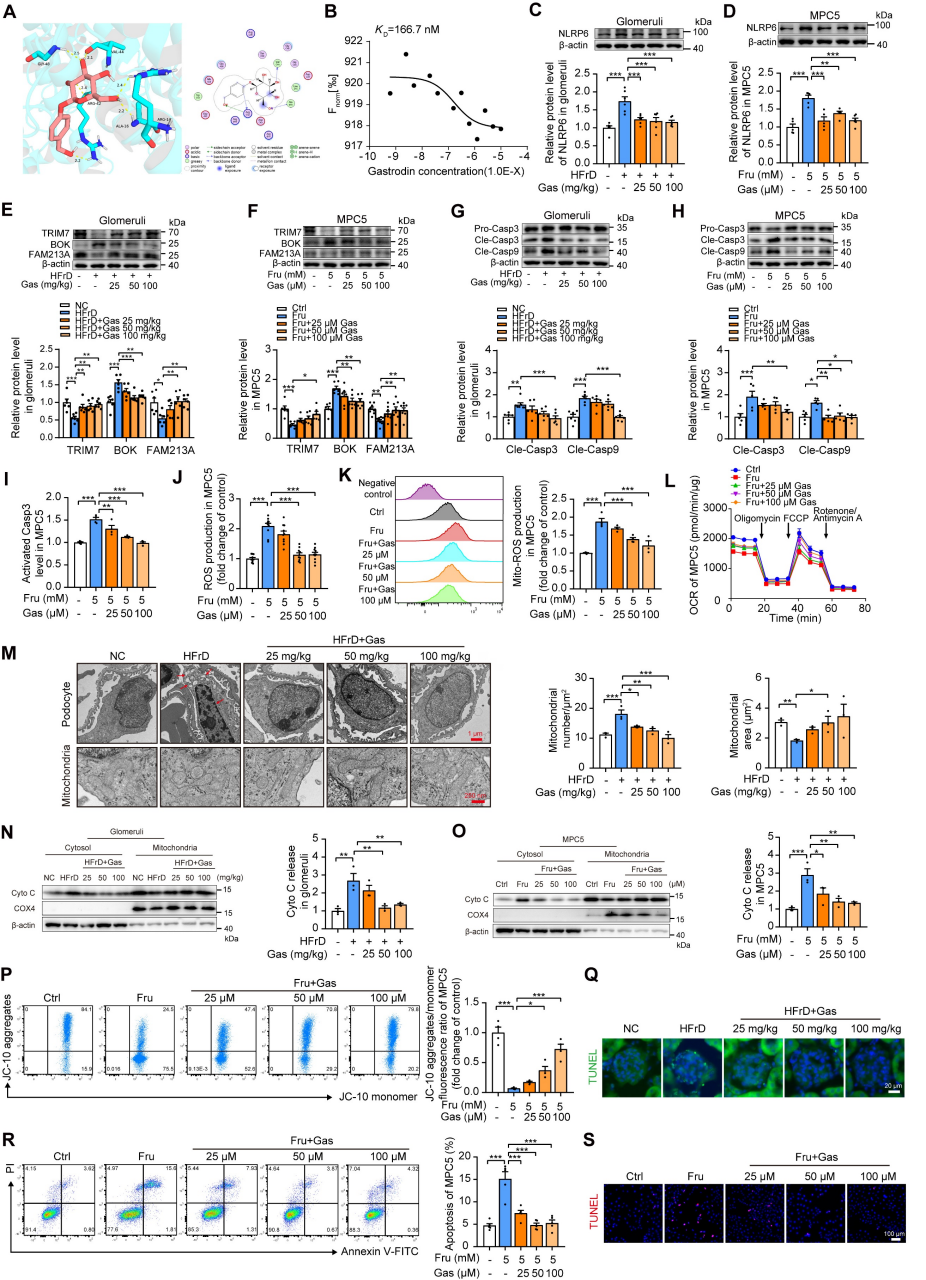
Pharmacological inhibition of NLRP6 by gastrodin ameliorates high fructose-induced glomerular podocyte mitochondria-mediated apoptosis. (A) Molecular docking shows interactions between gastrodin and NLRP6. The hydrogen-bonding interactions are shown in yellow dashed lines. (B) The binding affinity of gastrodin to NLRP6 was measured by MST, (*n* = 3). (C) Western blot detection of NLRP6 in control mouse and HFrD-fed mouse glomeruli with or without gastrodin treatment (25, 50, and 100 mg/kg), (*n* = 6). (D) Western blot detection of NLRP6 in MPC5, which was stimulated with 5 mM fructose as well as gastrodin (25, 50, and 100 μM) or not, (*n* = 5-6). (E) Western blot detection of TRIM7, BOK, and FAM213A in control mouse and HFrD-fed mouse glomeruli with or without gastrodin treatment (25, 50, and 100 mg/kg), (*n* = 6). (F) Western blot detection of TRIM7, BOK, and FAM213A in MPC5, which were stimulated with 5 mM fructose as well as gastrodin (25, 50, and 100 μM) or not, (*n* = 6-8). (G) Western blot detection of cleaved Caspase 3 and cleaved Caspase 9 in control mouse and HFrD-fed mouse glomeruli with or without gastrodin treatment (25, 50, and 100 mg/kg), (*n* = 6-7). (H) Western blot detection of cleaved Caspase 3 and cleaved Caspase 9 in MPC5, which were stimulated with 5 mM fructose as well as gastrodin (25, 50, and 100 μM) or not, (*n* = 5). (I) Flow cytometry analysis of activated Caspase 3 in MPC5, which were stimulated with 5 mM fructose as well as gastrodin (25, 50, and 100 μM) or not, (*n* = 4). (J) ROS levels were considered as the fluorescence intensity of fluorogenic probe DCFH_2_-DA, measured via microplate reader in MPC5. MPC5 were stimulated with 5 mM fructose as well as gastrodin (25, 50, and 100 μM) or not, (*n* = 10). (K) Mitochondrial ROS production was considered as the fluorescence intensity of fluorogenic probe MitoSOX, measured by flow cytometry in MPC5. MPC5 were stimulated with 5 mM fructose as well as gastrodin (25, 50, and 100 μM) or not, (*n* = 3-4). (L) OCR detection in MPC5, which were stimulated with 5 mM fructose as well as gastrodin (25, 50, and 100 μM) or not, (*n* = 8). (M) TEM images and quantification of podocyte in control mouse and HFrD-fed mouse glomeruli with or without gastrodin treatment (25, 50, and 100 mg/kg). Scale: 1 μm (Top), 250 nm (Bottom), (*n* = 3). (N) Western blot detection of Cyto C performed on mitochondrial and cytosolic fractions in control mouse and HFrD-fed mouse glomeruli with or without gastrodin treatment (25, 50, and 100 mg/kg), (*n* = 3). (O) Western blot detection of Cyto C was performed on mitochondrial and cytosolic fractions in MPC5, which were stimulated with 5 mM fructose as well as gastrodin (25, 50, and 100 μM) or not, (*n* = 3). (P) Flow cytometry analysis of mitochondrial membrane potentials using JC-10 dye in MPC5, which were stimulated with 5 mM fructose as well as gastrodin (25, 50, and 100 μM) or not, (*n* = 4). (Q) Representative IF images of TUNEL assay in control mouse and HFrD-fed mouse glomeruli with or without the treatment of gastrodin (25, 50, and 100 mg/kg), Scale: 20 μm. (R) Flow cytometry analysis of apoptotic cells through Annexin V-FITC/PI staining in MPC5, which were stimulated with 5 mM fructose as well as gastrodin (25, 50, and 100 μM) or not, (*n* = 5). (S) Representative IF images of TUNEL assay in MPC5, which were stimulated with 5 mM fructose as well as gastrodin (25, 50, and 100 μM) or not, Scale: 100 μm. Data are expressed as the mean ± SEM. ^*^P < 0.05, ^**^P < 0.01, ^***^P < 0.001.

Moreover, the identification of an effective NLRP6 inhibitor was preliminarily carried out. We initiated our investigation by employing molecular docking to assess the binding potential of gastrodin to NLRP6. The results of molecular docking analysis revealed a favorable binding energy of -7.176 Kcal/mol between gastrodin and NLRP6, indicating an interaction potential. This finding was further substantiated by the identification of specific active binding sites in pyrin domain (PYD), including Arg 14, Ala16, Arg 42, Val 44, and Gly 48 (Fig. 8A), which provided computational evidence for the direct interaction between gastrodin and NLRP6, potentially modulating its functional activity. To experimentally validate these computational predictions, we conducted MST experiment. Our MST analysis yielded a dissociation constant (KD) of 166.7 nM for the gastrodin-NLRP6 interaction (Fig. 8B). This low KD value confirmed the high binding affinity between gastrodin and NLRP6. Functional assays showed that this binding promoted NLRP6 protein degradation at the post-translational level, as evidenced by a significantly accelerated protein decay upon CHX treatment, without altering its mRNA expression (Fig. S14 A-B). Given these computational and experimental data, we hypothesized that gastrodin exerted its protective effect on high fructose-induced podocyte injury possibly through direct interaction with NLRP6, promoting its degradation.

Consistently, gastrodin significantly attenuated high fructose-induced overexpression of NLRP6 in both mouse glomeruli, MPC5, and HPCs (Fig. 8C-D and Fig. S15A). Gastrodin increased TRIM7 expression, decreased BOK expression, and then increased FAM213A expression in mouse glomeruli, MPC5, and HPCs with high fructose stimulation (Fig. 8E-F and Fig. S15B). Moreover, gastrodin inhibited Caspase 3 and Caspase 9 activation in *in vivo* and *in vitro* models (Fig. 8G-I and Fig. S15C-D). This compound blocked high fructose-induced ROS overproduction, and OCR decrease in MPC5 and HPCs (Fig. 8J-L and Fig. S15E-F). It ameliorated podocyte chromatin condensation, nuclear fragmentation, numerous vacuoles, as well as mitochondrial severe swelling, cristae loss, and vacuole formation in HFrD-fed mouse glomeruli (Fig. 8M). In addition, gastrodin significantly inhibited the leakage of Cyto C, mitochondrial depolarization as well as cell apoptosis increase in mouse glomeruli, MPC5, and HPCs under high fructose exposure (Fig. 8N-S and Fig. S15G-J).

To investigate the essential role of NLRP6 in protective effect of gastrodin on podocyte mitochondria-mediated apoptosis, the *Nlrp6-Flag* plasmid was used to increase NLRP6 expression, which was failed to be suppressed by gastrodin in MPC5 with high fructose exposure (Fig. S16A). *Nlrp6-Flag* blocked the interference of gastrodin on the abnormal expression of TRIM7, BOK, and FAM213A in high fructose-stimulated MPC5 (Fig. S16B). Of note, the rescuing impact of gastrodin on Caspase activation, ROS overproduction, OCR decrease, mitochondrial depolarization as well as Cyto C leakage was eliminated by NLRP6 overexpression in MPC5 under high fructose exposure (Fig. S16C-I). Additionally, NLRP6 overexpression abolished the inhibitory effect of gastrodin on high fructose-induced podocyte apoptosis (Fig. S16J-K). Taken together, these findings suggested that gastrodin inhibited NLRP6 and recovered the expression level of TRIM7, BOK as well as FAM213A, relieving high fructose-caused podocyte mitochondria-mediated apoptosis.

## Discussion

In this work, we observed abnormal expression of *Nlrp6* and *Nlrp12* in diverse clinical nephropathy samples, establishing their correlation with proteinuria or urine creatinine level. Then, we identified a significant enrichment of *Nlrp6* in mouse glomeruli compared with *Nlrp12*, and observed its higher expression in HPCs, MPC5 and PMPCs than that in other renal cortex cells with or without high fructose stimulation. The pronounced upregulation of *Nlrp6* was found in high fructose-induced glomerular podocyte injury, with disrupted cellular morphology and impaired kidney function. Notably, we found that *Nlrp6* deficiency significantly alleviated mitochondria-mediated apoptosis, being consistent with the attenuation of high fructose-induced glomerular podocyte injury. This protective effect was primarily related to enhanced TRIM7-driven *Bok* mRNA degradation and increased antioxidant activity of FAM213A. Thus, we underscored the potential of targeting NLRP6 suppression as a possible therapeutic strategy for managing podocyte injury associated with high fructose consumption, which was different from its conventional role in inflammatory signaling, establishing a novel non-inflammatory mechanism.

Abnormal expression of NLRPs is associated with kidney injury 34. We analyzed the expression of NLRPs in clinical samples obtained in GEO and GTEx databases, and observed that *Nlrp6* and *Nlrp12* were differentially expressed in both healthy volunteers and DN patients, suggesting the unique role for *Nlrp6* and *Nlrp12* in kidney. Both* Nlrp6* and *Nlrp12* are important in regulating innate immune homeostasis 35. However, *Nlrp12* deficiency accelerates colon cancer progression by activating non-classical NF-κB signaling 36, while NLRP6 suppresses excessive cytokine production by negatively regulating classical NF-κB pathway, and its deficiency increases cellular susceptibility to bacterial pathogens 17. To explore expression profiles of *Nlrp6* and *Nlrp12* in kidney physiology and pathology, we analyzed the expression of *Nlrp6* and *Nlrp12* in clinical kidney samples obtained in Nephroseq databases. Our results revealed that only *Nlrp6* mRNA levels were upregulated in glomeruli of nephrotic syndrome patients and correlated with occurrence of proteinuria and decreased creatinine level, suggesting that increased NLRP6 expression might aggravate clinical renal glomeruli injury.

Altered NLRP6 expression is frequently observed in various clinical diseases 37, 38. In patients with functional dyspepsia, low NLRP6 expression correlates with dysregulation of goblet cell homeostasis in duodenal spheroids 39. Similarly, hepatic NLRP6 expression is considerably decreased in liver biopsies of human non-alcoholic steatohepatitis patients 40, as well as in hepatocellular carcinoma patients with longer overall survival 41. However, a recent study has shown that NLRP6 inflammasome is activated in mouse kidney fibrosis induced by folic acid, whereas this activation is inhibited in human kidney proximal tubular epithelial cell apoptosis caused by rapamycin 42. Moreover, diminished expression of NLRP6 is observed in the kidney of patients with renal damage or mouse model of acute kidney injury, and its inhibition raises spontaneous cell apoptosis in serum-deprived mouse tubular cells 43. In addition, nicotine induces mild apoptosis alongside NLRP6 overexpression in human kidney proximal tubular epithelial cells 44. Here, we observed that podocytes had a higher level of NLRP6 expression than other renal cells under normal condition or high fructose stimulation, and the upregulation of NLRP6 in high fructose-stimulated mouse glomeruli, PMPCs, MPC5, and HPCs than control groups. These seemingly contradictory findings underscore that NLRP6 expression changes depend on the pathological context. For instance, in the liver, the downregulation of NLRP6 exacerbates the progression of non-alcoholic steatohepatitis and alcoholic hepatitis 40, 45. Conversely, in the context of certain infections, such as with the parasite Schistosoma mansoni, NLRP6 contributes to hepatic granuloma formation and aggravates injury 46, demonstrating that its role is inconsistent even in the same organ, which may depend on the pathogenic stimulus. Similarly, contradictory results regarding NLRP6 function exist in kidney pathology 43, 44. These observations underscore the complex and multifaceted function of NLRP6, prompting us to further investigate its contribution to kidney pathology.

Further RNA-seq analysis revealed that *Nlrp6* knockout markedly suppressed the apoptosis process in glomeruli of mice fed with HFrD, according to its highest enrichment score. Notably, the complement and coagulation cascade pathway, as well as the platelet pathway, with high *P* value, were enriched by the same differentially expressed genes *Fgg* and *Fga,* which encode fibrinogen. Fibrinogen, a protein mainly synthesized in the liver rather than the kidney, mediates the coagulation response 47. Hence, we did not conduct a deep investigation of these two pathways. Regarding the apoptosis process, *Nlrp6* deficiency suppressed Caspase 3 and Caspase 9 activation as well as mitochondrial depolarization, effectively mitigating high fructose-induced podocyte apoptosis. MOMP is recognized as a trigger of mitochondria-mediated apoptosis. Cyto C leakage as a hallmark of MOMP was inhibited by *Nlrp6* deficiency in mouse glomeruli and podocytes with high fructose stimulation. Interestingly, BOK, a vital inducer of MOMP, was downregulated by *Nlrp6* deficiency *in vivo* and *in vitro*. These findings demonstrated that NLRP6 inhibition may alleviate podocyte MOMP-associated apoptosis via a mitochondria-dependent pathway under high fructose simulation, and further investigation is warranted to elucidate the underlying mechanisms. For instance, podocyte-specific *Nlrp6* conditional knockout mice using Nephrin (NPHS1)-cre and Podocin (NPHS2)-cre lines are used to determine the direct effects of NLRP6 on podocyte apoptosis. And, the upstream mechanism by which high fructose triggers NLRP6 expression in podocytes remains unclear. Transcriptionally, high fructose modulates factors like peroxisome proliferator-activated receptor γ (PPARγ) and specificity protein 1 (SP1). The agonist of PPARγ is reported to induce NLRP6 upregulation in human epithelial colorectal adenocarcinoma cells 48. SP1 is elevated in high fructose-stimulated podocytes and directly transactivates the NLRP6 promoter in glioma cells 49, 50. Epigenetically, fructose alters substrates for DNA methylation and O-GlcNAcylation 51. NLRP6 is known to be increased via DNA hypomethylation in simian immunodeficiency virus infection 52, and it is activated by microRNA-152 in methamphetamine-induced brain pyroptosis 53. Post-translationally, NLRP6 is stabilized by cylindromatosis, which removes lysine 63-linked chains to suppress NLRP6 ubiquitination in intestinal inflammation 54. As fructose influences ubiquitination in metabolic diseases 55, similar deubiquitination of NLRP6 may occur in podocytes. Identifying the dominant pathway of how fructose causes high expression of NLPR6 will be a key future goal.

BOK maintains normal mitochondrial fusion, morphology, and bioenergetics, governing cell apoptosis pathway 56. *Bok* deficiency decreases proximity of endoplasmic reticulum to mitochondria and then suppresses Ca^2+^ transfer-induced apoptosis 57, while Ca^2+^ homeostasis related-calreticulin is positively correlated with decreased BOK protein level in stage II and III colorectal cancer patients 58. In this study, we found that BOK overexpression independently induced podocyte MOMP, while *Nlrp6* deficiency inhibited BOK to rescue high fructose-induced podocyte mitochondria-mediated apoptosis. However, the mechanism by which *Nlrp6* deficiency downregulates BOK remains unclear. A recent report has shown that mouse *Bok* mRNA possesses the major AU-rich element within its 3'UTR, possibly suppressing its expression by potent post-transcriptional mechanism 32. The TRIM family of proteins, which includes members with NHL or PRY/SPRY domains, exhibits RNA binding ability to control transcriptional and post-transcriptional RNA processes 59, 60. TRIM28, an RNA-binding protein, negatively regulates BOK stability through post-transcriptional modifications in human embryonic kidney 293T cells 32. Here, it is noteworthy that TRIM7 was identified as mediating the regulation of NLRP6 to BOK signaling in podocytes cultured with high fructose. *Nlrp6* deficiency increased the expression of TRIM7, enhancing its binding to the 3'UTR of *Bok* mRNA, thereby promoting *Bok* mRNA decay and relieving MOMP in podocytes under high fructose stimulation. These results highlighted the importance of increased TRIM7 expression in reducing *Bok* RNA stability, and inhibiting BOK as a critical link by which *Nlrp6* deficiency ameliorated podocyte mitochondria-mediated apoptosis under high fructose stimulation. In addition, our results showed that NLRP6 suppressed TRIM7 expression predominantly by promoting the degradation of its mRNA transcript. The mechanism by which NLRP6 negatively regulates TRIM7 may be of interest in future study, possibly being related to the intervention of N6-methyladenosine methylation on *Trim7*, based on recent studies as followed 61, 62. Modification motifs of m6A are present in the 3'UTR of *Trim7*, and its upregulation in patient osteosarcoma tissues has been attributed to loss of methyltransferase like 3/14-YTH domain-containing family protein 2-mediated m6A modification, which subsequently inhibits mRNA decay 61. Furthermore, in the mouse model of diabetes-induced dry eye disease, NLRP6 activation coincides with increased m6A modification of long noncoding RNA nuclear-enriched abundant transcript 1, exacerbating inflammation and corneal damage 62. Thus, NLRP6 may facilitate* Trim7* mRNA decay by actively orchestrating an increase in its m6A methylation status in high fructose-cultured podocytes, which should be further explored.

Although the association between BOK activation and mitochondria-mediated apoptosis has been proven in various cell types such as HCT116 cells treated with proteasome inhibitors 63, as well as MCF7 and H292 cells overexpressing BOK 64, 65, the direct mechanism in how BOK inhibition reduces apoptosis through mitochondria-mediated pathway in high fructose-exposed podocytes is still unknown. We investigated whether BOK triggers apoptosis by inducing an imbalance in Ca^2+^ homeostasis, and we found no significant change in the intracellular Ca^2+^ concentration of podocytes in the presence of *Nlrp6* deficiency or *Bok* deficiency, suggesting that NLRP6-driven apoptosis may not interfere with Ca^2+^ transfer. The burst of mitoROS and ensuing oxidative stress-induced mitochondrial dysfunction are known to contribute to cell apoptosis 66. In this study, we found that BOK overexpression caused an increase in ROS level in podocytes. Treatment with MitoTEMPO reduced podocyte mitoROS accumulation and apoptosis induced by BOK overexpression. Thus, low level of ROS might be a blocker of BOK-induced podocyte apoptosis. We next screened for a redox regulator controlled by BOK in podocytes, and an antioxidant protein FAM213A was emerged. Based on the structural similarity of FAM213A to peroxiredoxins and thioredoxins, it functions to decrease the accumulation of peroxides within the mitochondrial matrix 10. Change in the expression of FAM213A is considered to impact and predict the prognosis in patients with endometrial cancer, oral squamous cell carcinoma, or renal clear cell carcinoma 67-69. Indeed, we identified that BOK inhibition mediated FAM213A upregulation, which was promoted by *Nlrp6* deficiency in mouse glomeruli and podocytes under high fructose stimulation. Overexpression of FAM213A relieved BOK-induced oxidative stress and mitochondria-mediated apoptosis in podocytes. Therefore, *Nlrp6* deficiency improved TRIM7 expression, which in turn downregulated BOK, leading to increased FAM213A expression and decreased ROS production in high fructose-cultured podocytes. This cascade of events attenuated podocyte mitochondria-mediated apoptosis under high fructose exposure. Our work proposes that the suppression of NLRP6-mediated BOK/FAM213A pathway reduces ROS accumulation to relieve high fructose-induced podocyte apoptosis via the mitochondria-dependent pathway. However, it would be tempting for us to figure out the exact molecular mechanism by which BOK inhibits FAM213A, which may be related to transcriptional regulation in FAM213A 69.

The restoration of mitochondria function may be of vital importance for protecting against high fructose-induced podocyte apoptosis. Gastrodin, a phenolic glycoside found in *G. elata*, has shown promising therapeutic effects in traditional Chinese medicine for patients with kidney disorders 70. This herb lowers serum urea nitrogen and creatinine levels, and relieves the necrosis and swelling of glomeruli and proximal tubules in acetaminophen-induced kidney toxicity in rats 71. Gastrodin injection has been applied therapeutically to treat dizziness or vertigo sufferers 72. Gastrodin is reported to ameliorate cell apoptosis, possibly by inhibiting oxidative stress 73. This compound attenuates carbon tetrachloride-induced mouse kidney oxidative stress 13, increases antioxidant enzyme activity in ischemia-reperfusion injury-caused rat renal tubular cell apoptosis 15, and restores mitochondrial dysfunction in hydrogen peroxide-cultured rat myocardial cells 74. Here, we showed that gastrodin significantly improved mitochondrial fragmentation and dysfunction, decreased ROS accumulation, increased mitochondrial respiration, and ultimately ameliorated mitochondria-mediated apoptosis in glomerular podocytes exposed to high fructose, in accordance with its attenuation of podocyte injury. Further comprehensive investigation revealed that the anti-apoptotic effect of gastrodin was possibly attributed to its ability to bind with NLRP6 and decrease its expression, enhance the combination of TRIM7 with BOK, and increase FAM213A expression to inhibit high fructose-caused podocyte apoptosis through mitochondria-dependent pathway. Therefore, the suppression of NLRP6 by gastrodin can attenuate high fructose-induced podocyte mitochondria-mediated apoptosis. Gastrodin holds promise as a natural component for protecting podocytes against mitochondria-mediated apoptosis.

In summary, analysis of diverse clinical samples (blood and glomeruli) indicated a high correlation between NLRP6 and kidney injury compared with other NLRPs. Then, we demonstrated that NLRP6 aggravated glomerular podocyte injury induced by high fructose stimulation. Genetic knockout of *Nlrp6* prevented mitochondria-mediated apoptosis in podocyte injury. The treatment with gastrodin downregulated NLRP6 to prevent podocyte injury, at least partly by facilitating TRIM7-triggered *Bok* mRNA decay and improving FAM213A antioxidant effect. This study suggested that NLRP6 may be a potential therapeutic target for mitigating glomerular podocyte injury.

## Supplementary Material

Supplementary figures and tables.

## References

[1] Meliambro K, He JC, Campbell KN (2024). Podocyte-targeted therapies - progress and future directions. Nat Rev Nephrol.

[2] Shankland SJ, Jefferson JA, Wessely O (2023). Repurposing drugs for diseases associated with podocyte dysfunction. Kidney Int.

[3] Lin LR, Tan W, Pan XF, Tian E, Wu ZF, Yang JR (2022). Metabolic syndrome-related kidney injury: a review and update. Front Endocrinol (Lausanne).

[4] Li TS, Chen L, Wang SC, Yang YZ, Xu HJ, Gu HM (2019). Magnesium isoglycyrrhizinate ameliorates fructose-induced podocyte apoptosis through downregulation of miR-193a to increase WT1. Biochem Pharmacol.

[5] Fang L, Li TS, Zhang JZ, Liu ZH, Yang J, Wang BH (2021). Fructose drives mitochondrial metabolic reprogramming in podocytes via Hmgcs2-stimulated fatty acid degradation. Signal Transduct Target Ther.

[6] Liu SM, Yuan YY, Xue Y, Xing CY, Zhang B (2022). Podocyte injury in diabetic kidney disease: a focus on mitochondrial dysfunction. Front Cell Dev Biol.

[7] Kalkavan H, Green DR (2018). MOMP, cell suicide as a BCL-2 family business. Cell Death Differ.

[8] Shalaby R, Diwan A, Flores-Romero H, Hertlein V, Garcia-Saez AJ (2023). Visualization of BOK pores independent of BAX and BAK reveals a similar mechanism with differing regulation. Cell Death Differ.

[9] Wu WY, Wang ZX, Li TS, Ding XQ, Liu ZH, Yang J (2022). SSBP1 drives high fructose-induced glomerular podocyte ferroptosis via activating DNA-PK/p53 pathway. Redox Biol.

[10] Xu Y, Morse LR, da Silva RA, Odgren PR, Sasaki H, Stashenko P (2010). PAMM: a redox regulatory protein that modulates osteoclast differentiation. Antioxid Redox Signal.

[11] Chen YX, Yang H, Wang DS, Chen TT, Qi XL, Tao L (2024). Gastrodin alleviates mitochondrial dysfunction by regulating SIRT3-mediated TFAM acetylation in vascular dementia. Phytomedicine.

[12] Wu S, Huang R, Zhang RQ, Xiao C, Wang LL, Luo M (2023). Gastrodin and gastrodigenin improve energy metabolism disorders and mitochondrial dysfunction to antagonize vascular dementia. Molecules.

[13] Ma JQ, Sun YZ, Ming QL, Tian ZK, Zhang YJ, Liu CM (2020). Effects of gastrodin against carbon tetrachloride induced kidney inflammation and fibrosis in mice associated with the AMPK/Nrf2/HMGB1 pathway. Food Funct.

[14] Qiu CW, Chen B, Zhu HF, Liang YL, Mao LS (2024). Gastrodin alleviates cisplatin nephrotoxicity by inhibiting ferroptosis via the SIRT1/FOXO3A/GPX4 signaling pathway. J Ethnopharmacol.

[15] Zheng Y, Zhang N, Bai FD (2022). Gastrodin pretreatment alleviates renal ischemia-reperfusion injury. Urol Int.

[16] Weinberg SE, Sena LA, Chandel NS (2015). Mitochondria in the regulation of innate and adaptive immunity. Immunity.

[17] Anand PK, Malireddi RK, Lukens JR, Vogel P, Bertin J, Lamkanfi M (2012). NLRP6 negatively regulates innate immunity and host defence against bacterial pathogens. Nature.

[18] Wlodarska M, Thaiss CA, Nowarski R, Henao-Mejia J, Zhang JP, Brown EM (2014). NLRP6 inflammasome orchestrates the colonic host-microbial interface by regulating goblet cell mucus secretion. Cell.

[19] Levy M, Thaiss CA, Zeevi D, Dohnalová L, Zilberman-Schapira G, Mahdi JA (2015). Microbiota-modulated metabolites shape the intestinal microenvironment by regulating NLRP6 inflammasome signaling. Cell.

[20] Henao-Mejia J, Elinav E, Jin C, Hao L, Mehal WZ, Strowig T (2012). Inflammasome-mediated dysbiosis regulates progression of NAFLD and obesity. Nature.

[21] Ydens E, Demon D, Lornet G, De Winter V, Timmerman V, Lamkanfi M (2015). Nlrp6 promotes recovery after peripheral nerve injury independently of inflammasomes. J Neuroinflammation.

[22] Shen J, Xie P, Wang J, Yang F, Li S, Jiang H (2024). Nlrp6 protects from corticosterone-induced NSPC ferroptosis by modulating RIG-1/MAVS-mediated mitophagy. Redox Biol.

[23] Tang CF, Wang QN, Shen JY, Wang CY, Ding H, Wen SY (2023). Neuron stem cell NLRP6 sustains hippocampal neurogenesis to resist stress-induced depression. Acta Pharm Sin B.

[24] Fu J, Akat KM, Sun Z, Zhang WJ, Schlondorff D, Liu ZH (2019). Single-cell RNA profiling of glomerular cells shows dynamic changes in experimental diabetic kidney disease. J Am Soc Nephrol.

[25] Ding H, Tang CF, Wang W, Pan Y, Jiao RQ, Kong LD (2022). Polydatin ameliorates high fructose-induced podocyte oxidative stress via suppressing HIF-1α/NOX4 pathway. Pharmaceutics.

[26] Sampson MG, Robertson CC, Martini S, Mariani LH, Lemley KV, Gillies CE (2016). Integrative genomics identifies novel associations with APOL1 risk genotypes in black NEPTUNE subjects. J Am Soc Nephrol.

[27] Jiang XS, Chen XM, Hua W, He JL, Liu T, Li XJ (2020). PINK1/Parkin mediated mitophagy ameliorates palmitic acid-induced apoptosis through reducing mitochondrial ROS production in podocytes. Biochem Biophys Res Commun.

[28] Yuan Y, Wu YF, He MH, Jiang X (2024). Astragaloside IV protects against podocyte injury by upregulating mitophagy via the Mfn2/Pink1/Parkin Axis. Curr Mol Med.

[29] Gong P, Yue S, Shi FX, Yang WJ, Yao WB, Chen FX (2023). Protective effect of Astragaloside IV against cadmium-induced damage on mouse renal podocytes (MPC5). Molecules.

[30] Yang Y, Chen H, Huang SW, Chen H, Verkhratsky A, Niu JQ BOK-engaged mitophagy alleviates neuropathology in Alzheimer's disease. Brain. 2024: awae241.

[31] Liang X, Xiao J, Li XZC, Liu YJ, Lu Y, Wen YN (2022). A C-terminal glutamine recognition mechanism revealed by E3 ligase TRIM7 structures. Nat Chem Biol.

[32] Fernandez-Marrero Y, Bachmann D, Lauber E, Kaufmann T (2018). Negative regulation of BOK expression by recruitment of TRIM28 to regulatory elements in its 3' untranslated region. iScience.

[33] Garcia-Perez C, Roy SS, Naghdi S, Lin X, Davies E, Hajnóczky G (2012). Bid-induced mitochondrial membrane permeabilization waves propagated by local reactive oxygen species (ROS) signaling. Proc Natl Acad Sci USA.

[34] Anders HJ, Muruve DA (2011). The inflammasomes in kidney disease. J Am Soc Nephrol.

[35] Chen GY (2014). Role of Nlrp6 and Nlrp12 in the maintenance of intestinal homeostasis. Eur J Immunol.

[36] Allen IC, Wilson JE, Schneider M, Lich JD, Roberts RA, Arthur JC (2012). NLRP12 suppresses colon inflammation and tumorigenesis through the negative regulation of noncanonical NF-κB signaling. Immunity.

[37] Lin Y, Luo ZQ (2017). NLRP6 facilitates the interaction between TAB2/3 and TRIM38 in rheumatoid arthritis fibroblast-like synoviocytes. FEBS Lett.

[38] Chang LZ, Tian YY, Xu L, Hao QY, Song LY, Lu YY (2023). Spotlight on NLRP6 and tumor research situation: a potential cancer participant. J Immunol Res.

[39] Bruce JK, Burns GL, Sinn Soh W, Nair PM, Sherwin S, Fan K (2022). Defects in NLRP6, autophagy and goblet cell homeostasis are associated with reduced duodenal CRH receptor 2 expression in patients with functional dyspepsia. Brain Behav Immun.

[40] Huang CY, Liu QH, Tang Q, Jing XD, Wu T, Zhang JH (2021). Hepatocyte-specific deletion of Nlrp6 in mice exacerbates the development of non-alcoholic steatohepatitis. Free Radic Biol Med.

[41] Zhang L, Zhang XZ, Zhao W, Xiao XY, Liu SS, Peng Q (2022). NLRP6-dependent pyroptosis-related lncRNAs predict the prognosis of hepatocellular carcinoma. Front Med.

[42] Zheng CM, Lu KC, Chen YJ, Li CY, Lee YH, Chiu HW (2022). Matrix metalloproteinase-7 promotes chronic kidney disease progression via the induction of inflammasomes and the suppression of autophagy. Biomed Pharmacother.

[43] Valiño-Rivas L, Cuarental L, Nuñez G, Sanz AB, Ortiz A, Sanchez-Niño MD (2020). Loss of NLRP6 expression increases the severity of acute kidney injury. Nephrol Dial Transplant.

[44] Zheng CM, Lee YH, Chiu IJ, Chiu YJ, Sung LC, Hsu YH (2020). Nicotine causes nephrotoxicity through the induction of NLRP6 inflammasome and alpha7 nicotinic acetylcholine receptor. Toxics.

[45] Ji XY, Li LL, Lu PP, Li X, Tian DA, Liu M (2020). NLRP6 exerts a protective role via NF-kB with involvement of CCL20 in a mouse model of alcoholic hepatitis. Biochem Biophys Res Commun.

[46] Sanches RCO, Souza C, Marinho FV, Mambelli FS, Morais SB, Guimarães ES (2020). NLRP6 plays an important role in early hepatic immunopathology caused by schistosoma mansoni infection. Front Immunol.

[47] Sucajtys-Szulc E, Debska-Slizien A, Rutkowski B, Milczarek R, Szolkiewicz M, Swierczynski J (2023). Hepatocyte nuclear factor-1α increases fibrinogen gene expression in liver and plasma fibrinogen concentration in rats with experimental chronic renal failure. Int J Mol Sci.

[48] Kempster SL, Belteki G, Forhead AJ, Fowden AL, Catalano RD, Lam BY (2011). Developmental control of the Nlrp6 inflammasome and a substrate, IL-18, in mammalian intestine. Am J Physiol Gastrointest Liver Physiol.

[49] Yu Y, Cao F, Xiong Y, Zhou H (2021). SP1 transcriptionally activates NLRP6 inflammasome and induces immune evasion and radioresistance in glioma cells. Int Immunopharmacol.

[50] Zhou ZA, Wang YM, Xing Y, Pan SM, Wang WR, Yang J (2024). Magnolol inhibits high fructose-induced podocyte inflammation via downregulation of TKFC/Sp1/HDAC4/Notch1 activation. Pharmaceuticals (Basel).

[51] Tini G, Varma V, Lombardo R, Nolen GT, Lefebvre G, Descombes P (2020). DNA methylation during human adipogenesis and the impact of fructose. Genes Nutr.

[52] Premadasa LS, McDew-White M, Romero L, Gondo B, Drawec JA, Ling B (2025). Epigenetic modulation of the NLRP6 inflammasome sensor as a therapeutic modality to reduce necroptosis-driven gastrointestinal mucosal dysfunction in HIV/SIV infection. Cell Commun Signal.

[53] Oladapo A, Kannan M, Deshetty UM, Singh S, Buch S, Periyasamy P (2025). Methamphetamine-mediated astrocytic pyroptosis and neuroinflammation involves miR-152-NLRP6 inflammasome signaling axis. Redox Biol.

[54] Mukherjee S, Kumar R, Tsakem Lenou E, Basrur V, Kontoyiannis DL, Ioakeimidis F (2020). Deubiquitination of NLRP6 inflammasome by Cyld critically regulates intestinal inflammation. Nat Immunol.

[55] Rho H, Kim S, Kim SU, Kim JW, Lee SH, Park SH (2024). CHIP ameliorates nonalcoholic fatty liver disease via promoting K63- and K27-linked STX17 ubiquitination to facilitate autophagosome-lysosome fusion. Nat Commun.

[56] Schulman JJ, Szczesniak LM, Bunker EN, Nelson HA, Roe MW, Wagner LE 2nd (2019). Bok regulates mitochondrial fusion and morphology. Cell Death Differ.

[57] Carpio MA, Means RE, Brill AL, Sainz A, Ehrlich BE, Katz SG (2021). BOK controls apoptosis by Ca(2+) transfer through ER-mitochondrial contact sites. Cell Rep.

[58] Carberry S, D'Orsi B, Monsefi N, Salvucci M, Bacon O, Fay J (2018). The BAX/BAK-like protein BOK is a prognostic marker in colorectal cancer. Cell Death Dis.

[59] Goyani S, Roy M, Singh R (2021). TRIM-NHL as RNA binding ubiquitin E3 ligase (RBUL): implication in development and disease pathogenesis. Biochim Biophys Acta Mol Basis Dis.

[60] Williams FP, Haubrich K, Perez-Borrajero C, Hennig J (2019). Emerging RNA-binding roles in the TRIM family of ubiquitin ligases. Biol Chem.

[61] Zhou CL, Zhang ZC, Zhu XS, Qian GW, Zhou Y, Sun Y (2020). N6-Methyladenosine modification of the TRIM7 positively regulates tumorigenesis and chemoresistance in osteosarcoma through ubiquitination of BRMS1. EBioMedicine.

[62] Guo C, Yu MY, Liu JH, Jia Z, Liu H, Zhao SZ (2024). Molecular mechanism of Wilms tumour 1-associated protein in diabetes-related dry eye disease by mediating m6A methylation modification of lncRNA NEAT1. J Drug Target.

[63] Llambi F, Wang YM, Victor B, Yang M, Schneider DM, Gingras S (2016). BOK is a non-canonical BCL-2 family effector of apoptosis regulated by ER-associated degradation. Cell.

[64] Einsele-Scholz S, Malmsheimer S, Bertram K, Stehle D, Johänning J, Manz M (2016). Bok is a genuine multi-BH-domain protein that triggers apoptosis in the absence of Bax and Bak. J Cell Sci.

[65] Yang Y, Wu YJ, Meng XJ, Wang ZY, Younis M, Liu Y (2022). SARS-CoV-2 membrane protein causes the mitochondrial apoptosis and pulmonary edema via targeting BOK. Cell Death Differ.

[66] Su LJ, Zhang JH, Gomez H, Kellum JA, Peng ZY (2023). Mitochondria ROS and mitophagy in acute kidney injury. Autophagy.

[67] Ren XT, Ma L, Wang N, Zhou RN, Wu JH, Xie X (2021). Antioxidant gene signature impacts the immune infiltration and predicts the prognosis of kidney renal clear cell carcinoma. Front Genet.

[68] Yu J, Yao HW, Liu TT, Wang D, Shi JH, Yuan GW (2022). Comprehensive analysis and experimental validation of a novel estrogen/progesterone-related prognostic signature for endometrial cancer. J Pers Med.

[69] Chen YF, Wei YY, Yang CC, Liu CJ, Yeh LY, Chou CH (2019). miR-125b suppresses oral oncogenicity by targeting the anti-oxidative gene PRXL2A. Redox Biol.

[70] Song GM (2018). Clinical observation on Banxia Baizhu Tianma Decoction combined with western medicine in the treatment of hypertension. Guangming Journal of Chinese Medicine.

[71] Seok PR, Kim JH, Kwon HR, Heo JS, Choi JR, Shin JH (2018). Protective effects of Gastrodia elata Blume on acetaminophen-induced liver and kidney toxicity in rats. Food Sci Biotechnol.

[72] Lai YF, Wang RN, Li W, Zhu H, Fei SY, Shi HH (2022). Clinical and economic analysis of Gastrodin injection for dizziness or vertigo: a retrospective cohort study based on electronic health records in China. Chin Med.

[73] Lin JL, Shi YF, Miao JS, Wu YH, Lin H, Wu JW (2019). Gastrodin alleviates oxidative stress-induced apoptosis and cellular dysfunction in human umbilical vein endothelial cells via the nuclear factor-erythroid 2-related factor 2/heme oxygenase-1 pathway and accelerates wound healing in vivo. Front Pharmacol.

[74] Cheng QQ, Wan YW, Yang WM, Tian MH, Wang YC, He HY (2020). Gastrodin protects H9c2 cardiomyocytes against oxidative injury by ameliorating imbalanced mitochondrial dynamics and mitochondrial dysfunction. Acta Pharmacol Sin.

