# Supplementary material for: Gastrodin alleviates high fructose-induced podocyte mitochondria-mediated apoptosis by inhibiting NLRP6 to facilitate TRIM7-triggered Bok mRNA degradation
Source: Int J Biol Sci. 2026 Jan 1;22(3):1162–85. doi: 10.7150/ijbs.120307 (PMC12836499; doi:10.7150/ijbs.120307)
Supplement: Supplementary file 1 — Supplementary figures and tables. [file ijbsv22p1162s1.pdf]

Supplementary material

Gastrodin alleviates high fructose-induced podocyte mitochondria-mediated apoptosis by inhibiting NLRP6 to facilitate TRIM7-triggered *Bok* mRNA degradation

Hong Ding, Wen-Xuan Wang, Qiong-Dan Liang, Chuan-Feng Tang, Tang-Di Xu, Zi-An Miao, Bang-Xing Han, Ling-Dong Kong

Correspondence to: hanbx1978@sina.com or kongld@nju.edu.cn

This PDF file includes:

Figure S1 to S16
Table S1 to S3

Figure S1

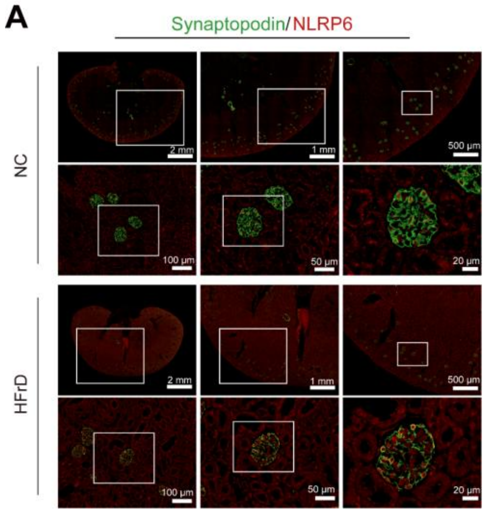

Fig. S1 The location of NLRP6 in the mouse kidney. (A) Representative IF images of Synaptopodin (green) and NLRP6 (red) in glomeruli from the whole kidney of WT mouse with or without HFrD, (*n* = 3).

Figure S2

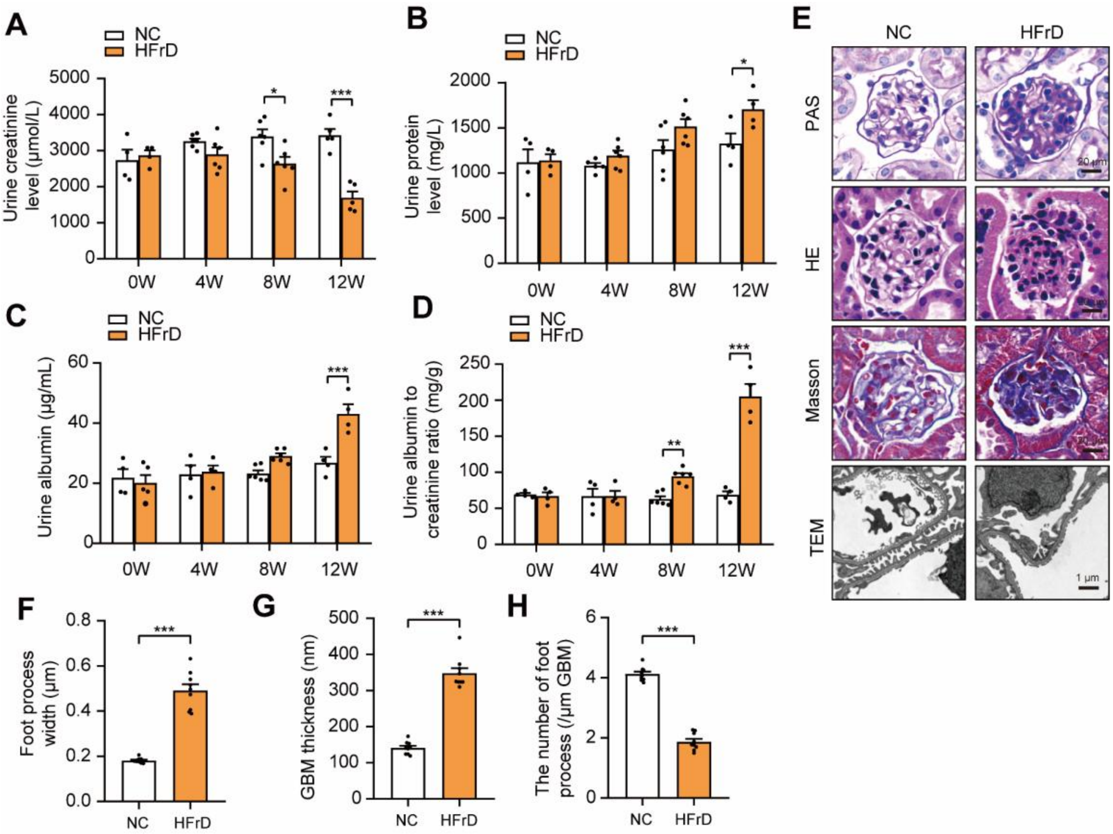

Fig. S2. High fructose diet induces mouse glomerular podocyte injury. (A-D) Urine creatinine, urine protein, urine albumin, and urine albumin to creatinine ratio were measured in WT mice with or without HFrD, (*n* = 4-6). (E-H) Representative images show glomerular changes by morphological examinations, including PAS, HE, and Masson staining and TEM analysis in WT mice with or without HFrD. Scale: PAS, HE, Masson: 20 μm; TEM: 1 μm, (*n* = 9). Data are expressed as the mean ± SEM. $^{*}P < 0.05$, $^{**}P < 0.01$, $^{***}P < 0.001$.

Figure S3

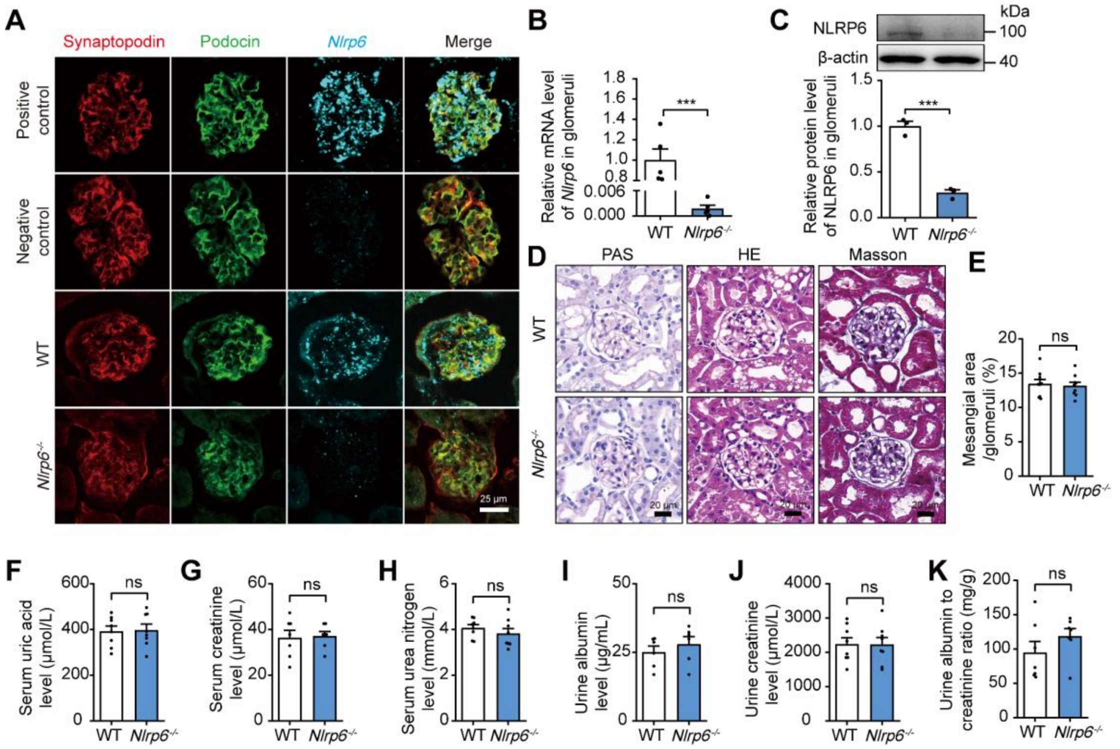

Fig. S3. Phenotypic identification and renal function assay in *Nlrp6* knockout mice. (A) The representative micrographs of RNAscope (*Nlrp6* mRNA, cyan) and IF (Podocin, green; Synaptopodin, red) co-labeled podocytes of WT or *Nlrp6*$^{-/-}$ mouse glomeruli. Scale: 25 μm. (B) *Nlrp6* mRNA level in WT and *Nlrp6*$^{-/-}$ mouse glomeruli, (*n* = 5). (C) Western blot detection of NLRP6 in WT or *Nlrp6*$^{-/-}$ mouse glomeruli, (*n* = 3). (D-E) Representative images show glomerular changes by morphological examinations, including PAS, HE, and Masson staining in WT and *Nlrp6*$^{-/-}$ mice. Scale: PAS, HE, Masson: 20 μm. (F-K) Serum uric acid, creatinine, urea nitrogen, as well as urine albumin, creatinine, and the ratio of urine albumin to creatinine were measured in WT and *Nlrp6*$^{-/-}$ mouse, (*n* = 6-8), respectively. Data are expressed as the mean ± SEM. $^{***}P < 0.001$. no significance, ns.

Figure S4

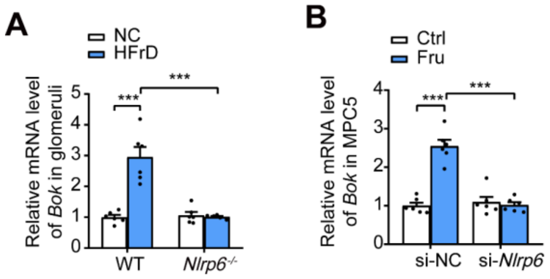

Fig. S4. *Bok* mRNA level was decreased by NLRP6 inhibition in high fructose-stimulated mice glomeruli and podocytes. (A) *Bok* mRNA level in WT and *Nlrp6*$^{-/-}$ mouse glomeruli with or without HFrD, (n = 5-6). (B) *Bok* mRNA level in MPC5, which were stimulated with or without 5 mM fructose after being transfected with siRNA-NC or siRNA-*Nlrp6*, (n = 6). Data are expressed as the mean ± SEM. $^{***}P < 0.001$.

Figure S5

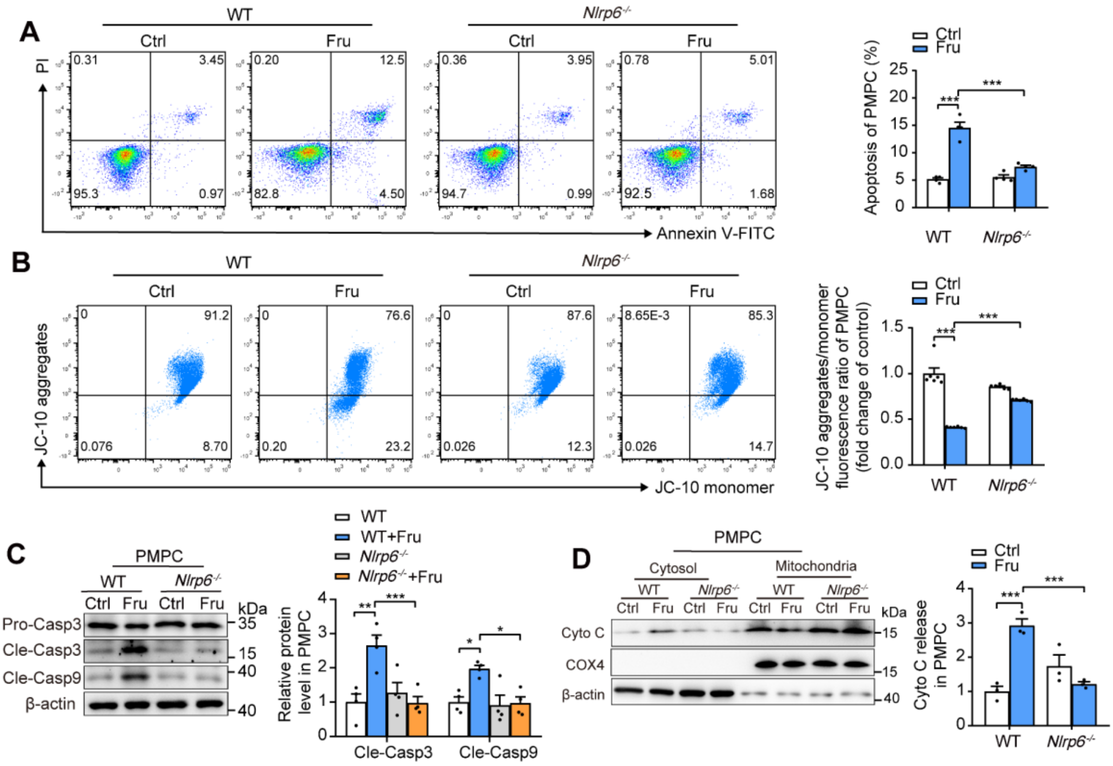

Fig. S5. NLRP6 inhibition attenuates mitochondria-mediated apoptosis in PMPCs under high fructose stimulation. (A) Flow cytometry analysis of apoptotic cells through Annexin V-FITC/PI staining. PMPCs isolated from WT and *Nlrp6*$^{-/-}$ mice were stimulated with or without 5 mM fructose, (n = 6). (B) Flow cytometry analysis of mitochondrial membrane potentials using JC-10 dye. PMPCs isolated from WT and *Nlrp6*$^{-/-}$ mice were stimulated with or without 5 mM fructose, (n = 6). (C) Western blot detection of cleaved Caspase 3 and cleaved Caspase 9 in PMPCs isolated from WT and *Nlrp6*$^{-/-}$ mice, which were stimulated with or without 5 mM fructose, (n = 4). (D) Western blot detection of Cyto C was performed on mitochondrial and cytosolic fractions in PMPCs isolated from WT and *Nlrp6*$^{-/-}$ mice, which were stimulated with or without 5 mM fructose, (n = 3). Data are expressed as the mean ± SEM. $^{*}P < 0.05$, $^{**}P < 0.01$, $^{***}P < 0.001$.

Figure S6

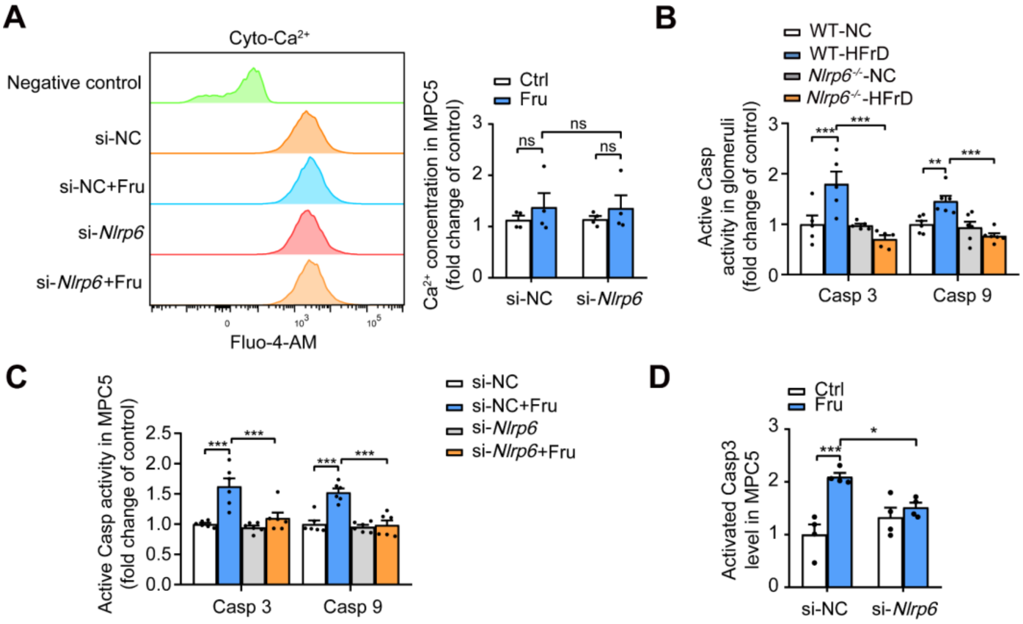

Fig. S6. *Nlrp6* deficiency reduces high fructose-induced high enzyme activity of Caspase 3 and Caspase 9 without regulating Ca^{2+} flux in glomeruli podocytes. (A) Flow cytometry analysis of total intracellular Ca^{2+} concentration using Fluo-4 AM (1 μM) in MPC5, which were stimulated with or without 5 mM fructose after being transfected with siRNA-NC or siRNA-*Nlrp6*, (n = 4). (B) Activities of Caspase 3 and Caspase 9 were examined in WT and *Nlrp6*$^{-/-}$ mouse glomeruli with or without HFrD, (n = 5-6). (C) Activities of Caspase 3 and Caspase 9 were examined in MPC5, which were stimulated with or without 5 mM fructose after being transfected with siRNA-NC or siRNA-*Nlrp6*, (n = 6). (D) Flow cytometry analysis of activated Caspase 3 MPC5, which were stimulated with or without 5 mM fructose after being transfected with siRNA-NC or siRNA-*Nlrp6*, (n = 4). Data are expressed as the mean ± SEM. $^{*}P < 0.05$, $^{**}P < 0.01$, $^{***}P < 0.001$. no significance, ns.

Figure S7

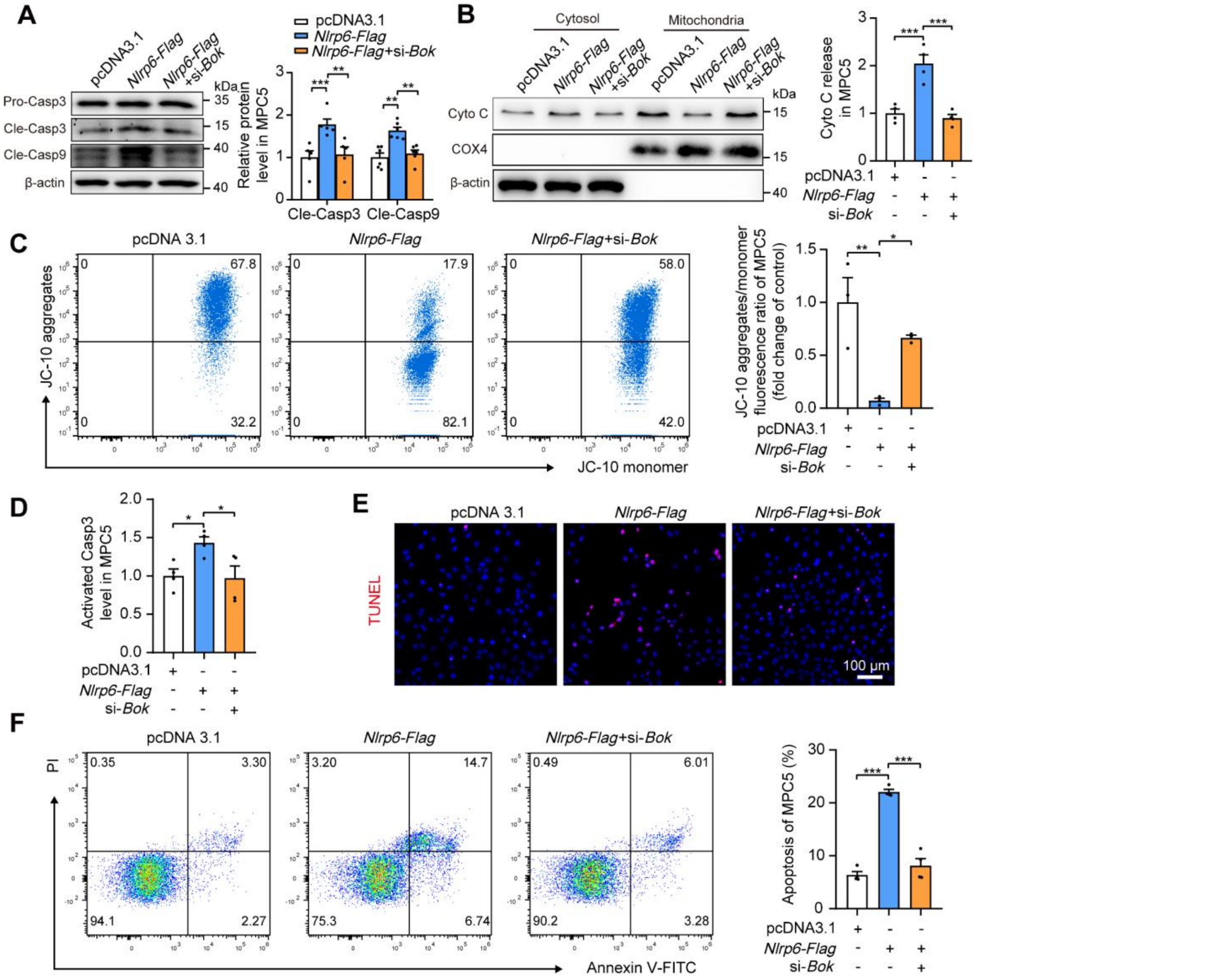

Fig. S7. Downregulated BOK relieves NLRP6-induced mitochondria-mediated apoptosis in podocytes. (A) Western blot detection of cleaved Caspase 3 and cleaved Caspase 9 in MPC5, which were transfected with *Nlrp6-Flag* or siRNA-*Bok*, (*n* = 6). (B) Western blot detection of Cyto C was performed on mitochondrial or cytosolic fractions in MPC5, which were transfected with *Nlrp6-Flag* or siRNA-*Bok*, (*n* = 4). (C) Flow cytometry analysis of mitochondrial membrane potentials using JC-10 dye in MPC5, which were transfected with *Nlrp6-Flag* or siRNA-*Bok*, (*n* = 3). (D) Flow cytometry analysis of activated Caspase 3 MPC5, which were transfected with *Nlrp6-Flag* or siRNA-*Bok*, (*n* = 4). (E) Representative IF images of TUNEL assay in MPC5, which were transfected with *Nlrp6-Flag* or siRNA-*Bok*, Scale: 100 μm. (F) Flow cytometry analysis of apoptotic cells through Annexin V-FITC/PI staining. MPC5 were transfected with *Nlrp6-Flag* or siRNA-*Bok*, (*n* = 4). Data are expressed as the mean ± SEM. $^{*}P < 0.05$, $^{**}P < 0.01$, $^{***}P < 0.001$.

Figure S8

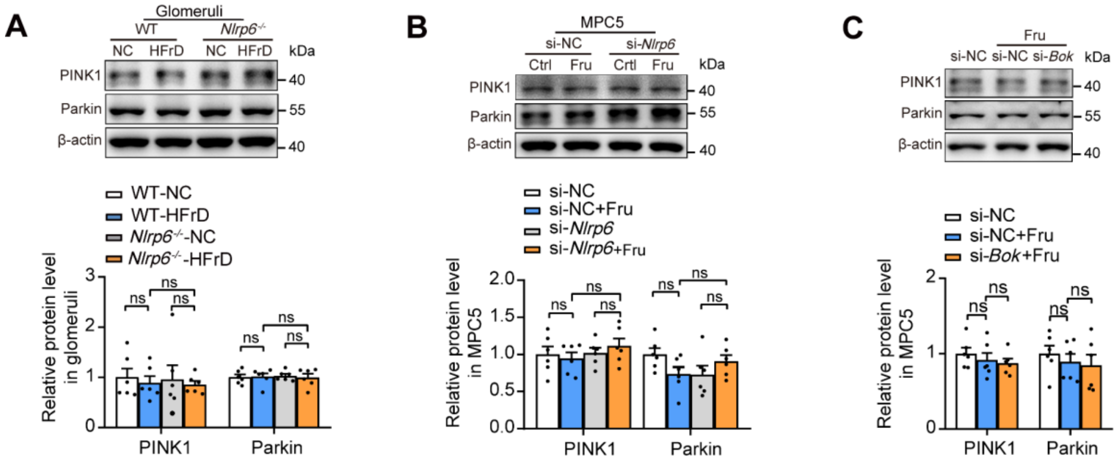

Fig. S8. NLRP6 and BOK do not interfere with PINK1/Parkin-mediated mitophagy in high fructose-stimulated mice glomeruli and podocytes. (A) Western blot detection of PINK1 and Parkin in WT and *Nlrp6*$^{-/-}$ mouse glomeruli with or without HFrD, (n = 6). (B) Western blot detection of PINK1 and Parkin in MPC5, which were transfected with siRNA-NC or siRNA-*Nlrp6*, subsequently stimulated with 5 mM fructose or not, (n = 6). (C) Western blot detection of PINK1 and Parkin in MPC5, which were stimulated with or without 5 mM fructose after being transfected with siRNA-NC or siRNA-*Bok*, (n = 6). Data are expressed as the mean ± SEM, no significance, ns.

Figure S9

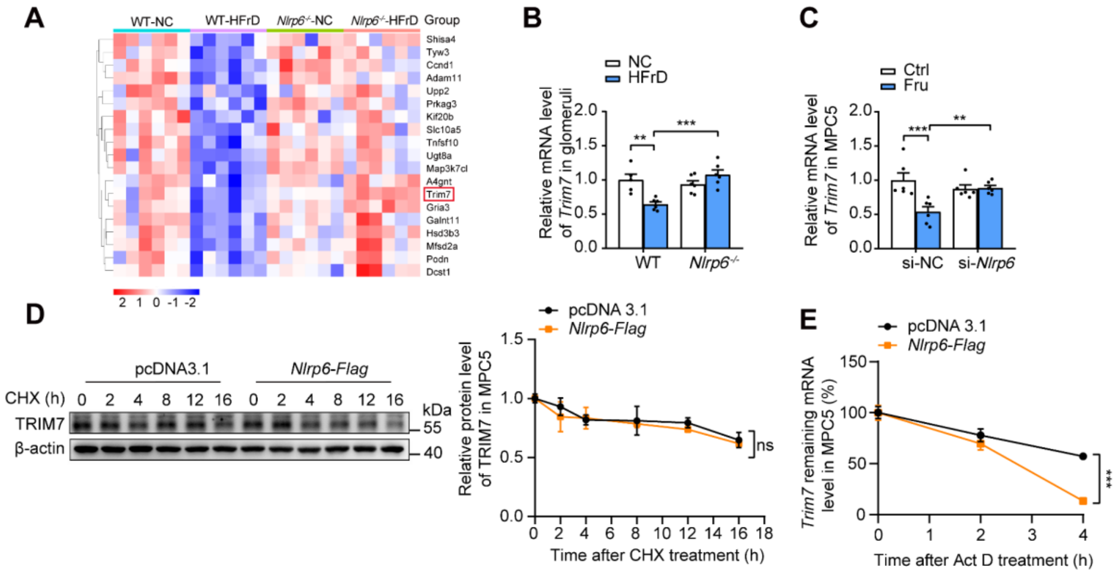

Fig. S9. *Trim7* mRNA level was decreased in high fructose-stimulated mice glomeruli and podocytes. (A) Heatmap showed genes that were downregulated in WT-HFrD versus WT-NC but upregulated in *Nlrp6*$^{-/-}$-HFrD versus WT-HFrD from the data of RNA-seq analysis of mouse-isolated glomeruli, (n = 6). (B) *Trim7* mRNA level in WT and *Nlrp6*$^{-/-}$ mouse glomeruli with or without HFrD, (n = 5-6). (C) *Trim7* mRNA level in MPC5, which were stimulated with or without 5 mM fructose after being transfected with siRNA-NC or siRNA-*Nlrp6*, (n = 6). (D) Western blot detection of TRIM7 in MPC5 exposed to 5 μg/mL CHX or not for different times after being transfected with vector or *Nlrp6-Flag*, (n = 4). (E) *Trim7* remaining mRNA level in MPC5 exposed to 5 μg/mL Act D or not for different times after being transfected with vector or *Nlrp6-Flag*, (n = 4). Data are expressed as the mean ± SEM. $^{**}P < 0.01$, $^{***}P < 0.001$. no significance, ns.

Figure S10

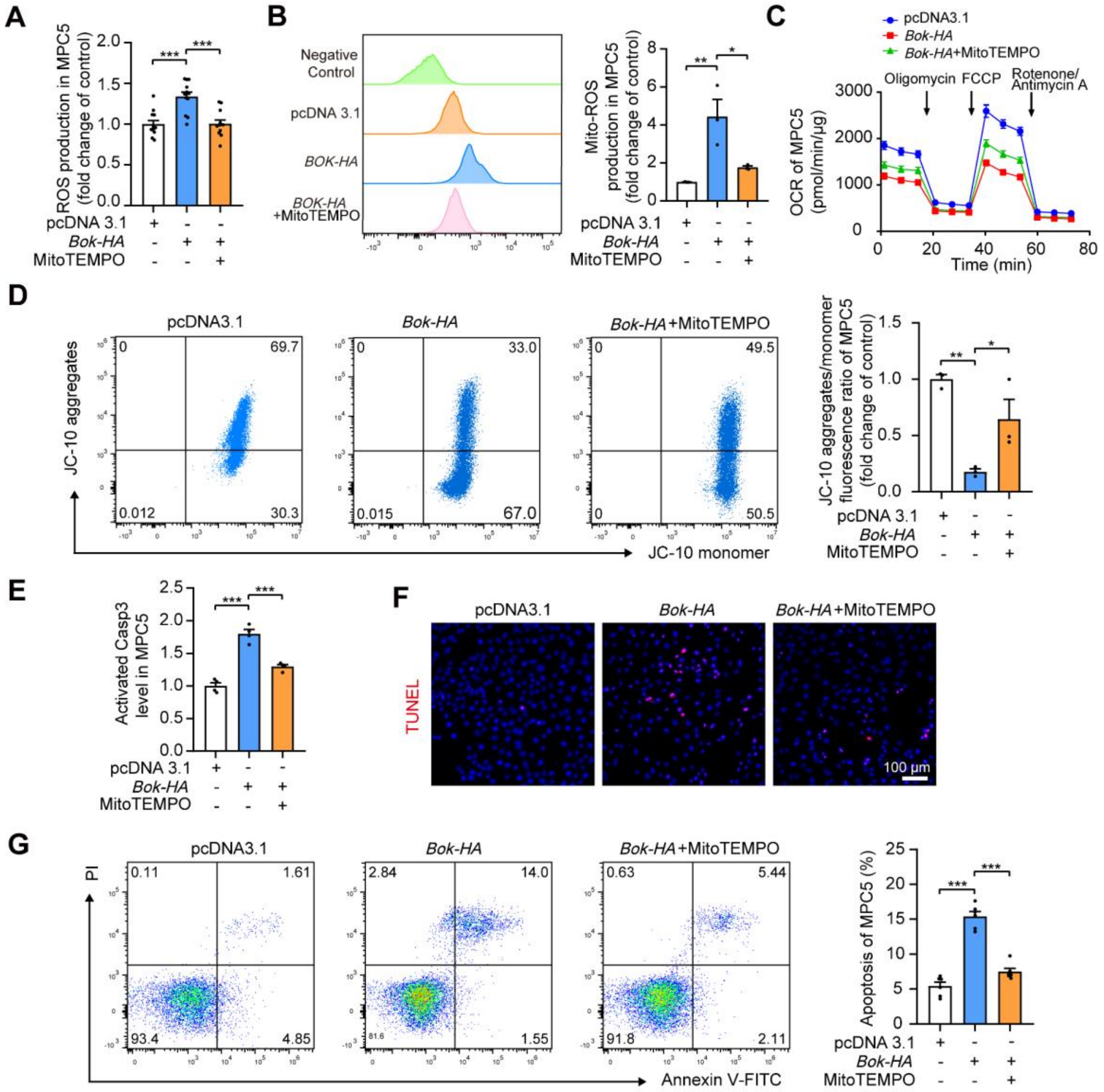

Fig. S10. The overexpression of BOK causes podocyte ROS accumulation and mitochondria-mediated apoptosis. (A) ROS levels were considered as the fluorescence intensity of fluorogenic probe DCFH$_2$-DA, measured via microplate reader in MPC5, which were pretreated with or without 10 μM MitoTEMPO before being transfected with vector or *Bok-HA*, (*n* = 12). (B) Mitochondrial ROS production was considered as the fluorescence intensity of labeling fluorogenic probe MitoSOX, measured by flow cytometry in MPC5, which were pretreated with or without 10 μM MitoTEMPO before being transfected with vector or *Bok-HA*, (*n* = 3). (C) OCR detection in MPC5, which was pretreated with or without 10 μM MitoTEMPO before being transfected with vector or *Bok-HA*, (*n* = 5-6). (D) Flow cytometry analysis of mitochondrial membrane potentials using JC-10 dye in MPC5, which were pretreated with or without 10 μM MitoTEMPO before being transfected with vector or *Bok-HA*, (*n* = 3). (E) Flow cytometry analysis of activated Caspase 3 in MPC5, which was pretreated with or without 10 μM MitoTEMPO before being transfected with vector or *Bok-HA*, (*n* = 4). (F) Representative IF images of TUNEL assay in MPC5, which was pretreated with or without 10 μM MitoTEMPO before being transfected with vector or *Bok-HA*, Scale: 100 μm. (G) Flow cytometry analysis of the percentage of apoptotic cells through Annexin V-FITC/PI staining. MPC5 were pretreated with or without 10 μM MitoTEMPO before being transfected with vector or *Bok-HA*, (*n* = 6). Data are expressed as the mean ± SEM. $^{*}P < 0.05$, $^{**}P < 0.01$, $^{***}P < 0.001$.

Figure S11

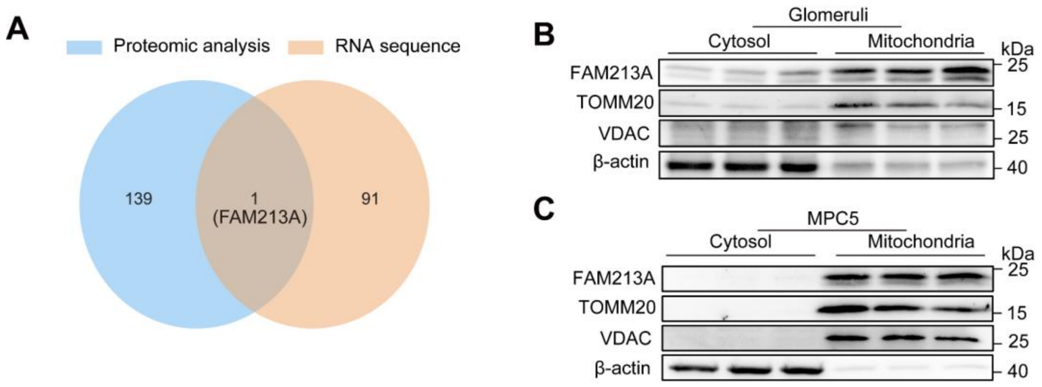

Fig. S11. Antioxidant protein FAM213A is mainly expressed in glomerular podocyte mitochondria. (A) Compared with normal chow, overlapped the differentially expressed genes (downregulated) of rat glomeruli with 10% fructose solution (W/V) for 12 weeks between Proteomic analysis and RNA-seq analysis. (B) Western blot detection of FAM213A, TOMM20, and VDAC performed on mitochondrial and cytosolic fractions in WT mouse glomeruli, (n = 3). (C) Western blot detection of FAM213A, TOMM20, and VDAC was performed on mitochondrial and cytosolic fractions in MPC5, (n = 3). VDAC: voltage-dependent anion channel.

Figure S12

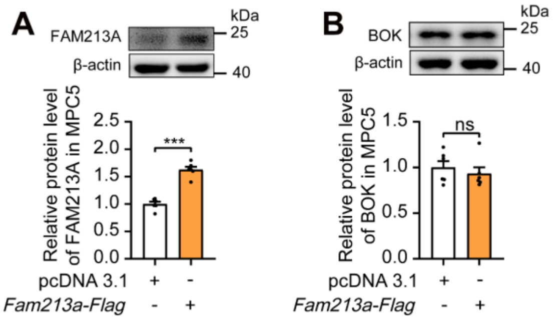

Fig. S12. The influence of *Fam213a-Flag* transfection on expression of FAM213A and BOK in podocytes. (A) Western blot detection of FAM213A in MPC5 transfected with vector or *Fam213a-Flag*, (n = 6). (B) Western blot detection of BOK in MPC5 transfected with vector or *Fam213a-Flag*, (n = 6). Data are expressed as the mean ± SEM. $^{***}P < 0.001$. no significance, ns.

Figure S13

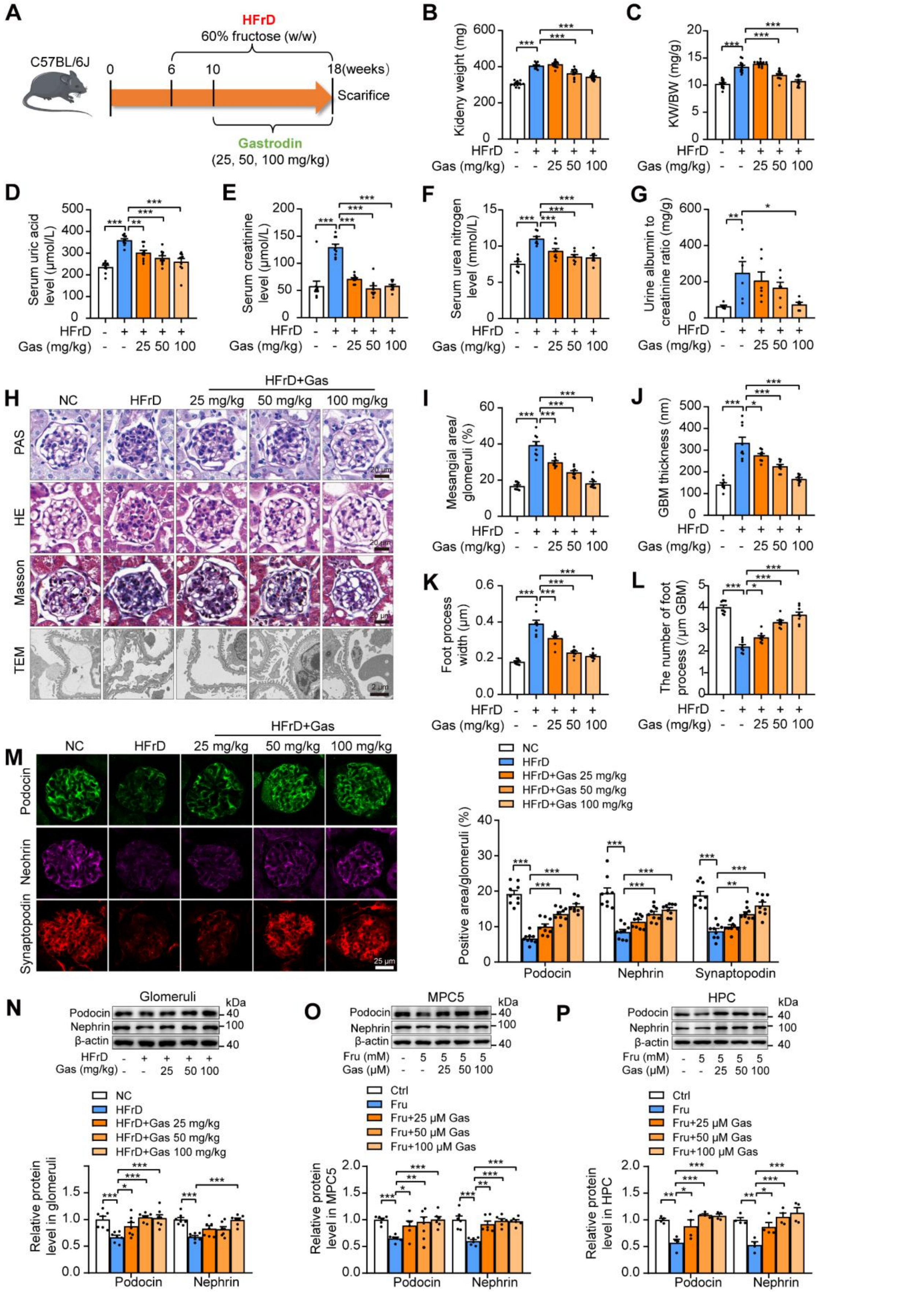

Fig. S13. Gastrodin prevents high fructose-induced glomerular podocyte injury. (A) A diagram of the procedure of HFrD-fed mouse treated with gastrodin. (B-C) The kidney wet weight, and kidney index of

control mouse and HFrD-fed mouse with or without gastrodin treatment (25, 50, and 100 mg/kg), (n = 12). (D-G) Serum uric acid, creatinine, urea nitrogen, and the ratio of urine albumin to creatinine were measured in control mice and HFrD-fed mice with or without gastrodin treatment (25, 50, and 100 mg/kg), (n = 6-10). (H-L) Representative images show glomerular changes by morphological examinations, including PAS staining, HE staining, Masson staining, and TEM analysis in control mice and HFrD-fed mice with or without gastrodin treatment (25, 50, and 100 mg/kg). Scale: PAS, HE, Masson: 20 μm; TEM: 2 μm, (n = 9). (M) Representative images of IF and quantifications of Podocin (green), Nephrin (magenta), and Synaptopodin (red) in glomeruli from control mice and HFrD-fed mice with or without gastrodin treatment (25, 50, and 100 mg/kg). Scale: 25 μm, (n = 9). (N) Western blot detection of Podocin and Nephrin in control mouse and HFrD-fed mouse glomeruli with or without gastrodin treatment (25, 50, and 100 mg/kg), (n = 7). (O) Western blot detection of Podocin and Nephrin expression in MPC5, which were stimulated with 5 mM fructose as well as gastrodin (25, 50, and 100 μM) or not, (n = 6). (P) Western blot detection of Podocin and Nephrin expression in HPCs, which were stimulated with 5 mM fructose as well as gastrodin (25, 50, and 100 μM) or not, (n = 6). Gas: gastrodin. Data are expressed as the mean ± SEM. $^{*}P < 0.05$, $^{**}P < 0.01$, $^{***}P < 0.001$.

Figure S14

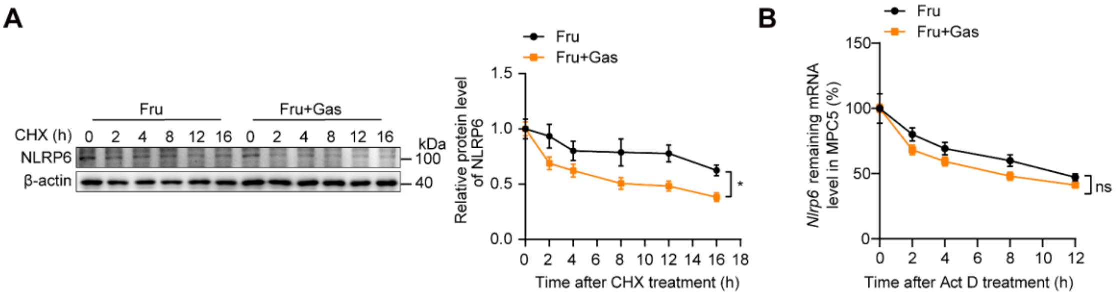

Fig. S14. Gastrodin promotes the degradation of NLRP6 protein in MPC5. (A) Western blot detection of NLRP6 in MPC5 exposed to 5 μg/mL CHX or not for different times after being cultured with 5 mM fructose as well as 100 μM gastrodin or not, (n = 4). (B) *Nlrp6* remaining mRNA level in MPC5 exposed to 5 μg/mL Act D or not for different times after being cultured with 5 mM fructose as well as 100 μM gastrodin or not, (n = 6). Data are expressed as the mean ± SEM. $^{*}P < 0.05$. no significance, ns.

Figure S15

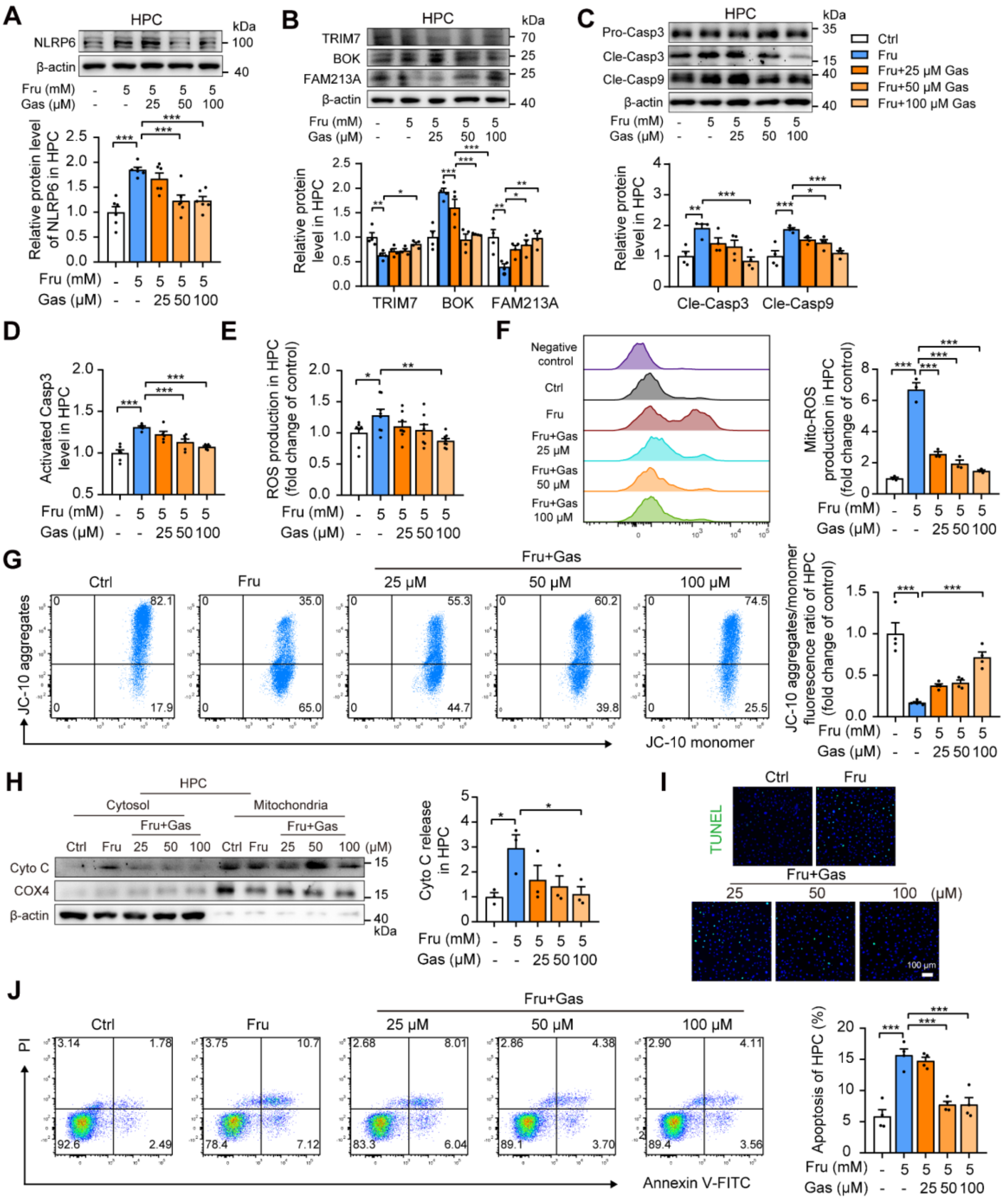

Fig. S15. Pharmacological inhibition of NLRP6 by gastrodin ameliorates high fructose-induced mitochondria-mediated apoptosis in HPCs. (A) Western blot detection of NLRP6 in HPCs, which were stimulated with 5 mM fructose as well as gastrodin (25, 50, and 100 μM) or not, (n = 6). (B) Western blot detection of TRIM7, BOK, and FAM213A in HPCs, which were stimulated with 5 mM fructose as well as gastrodin (25, 50, and 100 μM) or not, (n = 4). (C) Western blot detection of cleaved Caspase 3 and cleaved Caspase 9 in HPCs, which were stimulated with 5 mM fructose as well as gastrodin (25, 50, and 100 μM) or not, (n = 4). (D) Flow cytometry analysis of activated Caspase 3 in HPCs, which were stimulated with 5 mM fructose as well as gastrodin (25, 50, and 100 μM) or not, (n = 6). (E) ROS levels were considered as the fluorescence intensity of fluorogenic probe DCFH$_2$-DA, measured via microplate reader in HPCs. HPCs were stimulated with 5 mM fructose as well as gastrodin (25, 50, and 100 μM) or not, (n = 10). (F) Mitochondrial ROS production was considered as the fluorescence intensity of fluorogenic probe MitoSOX, measured by flow cytometry. HPCs were stimulated with 5 mM fructose as well as gastrodin (25, 50, and 100 μM) or not, (n = 3). (G) Flow cytometry analysis of mitochondrial membrane potentials using JC-10 dye in HPCs, which were stimulated

with 5 mM fructose as well as gastrodin (25, 50, and 100 μM) or not, (n = 4). (H) Western blot detection of Cyto C was performed on mitochondrial and cytosolic fractions in HPCs, which were stimulated with 5 mM fructose as well as gastrodin (25, 50, and 100 μM) or not, (n = 3). (I) Representative IF images of TUNEL assay in HPCs, which were stimulated with 5 mM fructose as well as gastrodin (25, 50, and 100 μM) or not, Scale: 100 μm. (J) Flow cytometry analysis of apoptotic cells through Annexin V-FITC/PI staining in HPCs, which were stimulated with 5 mM fructose as well as gastrodin (25, 50, and 100 μM) or not, (n = 4). Data are expressed as the mean ± SEM. $^{*}P < 0.05$, $^{**}P < 0.01$, $^{***}P < 0.001$.

Figure S16

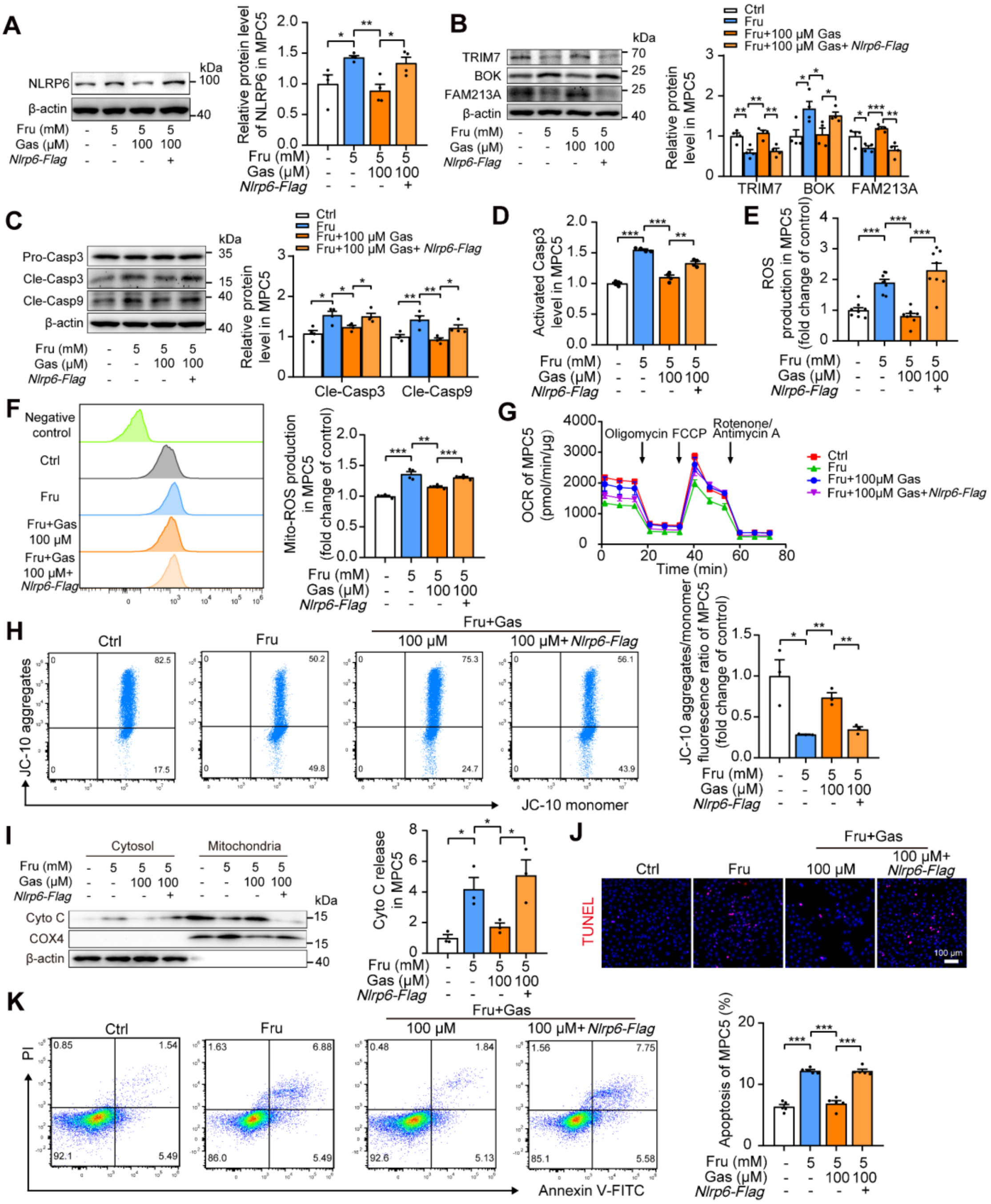

Fig. S16. NLRP6 overexpression blocked gastrodin protective effect on podocyte mitochondria-mediated apoptosis with high fructose exposure. (A) Western blot detection of NLRP6 in MPC5 transfected with *Nlrp6-Flag* plasmid or not, and then exposed with 5 mM fructose as well as 100 μM gastrodin or not, (*n* = 4). (B) Western blot detection of TRIM7, BOK, and FAM213A in MPC5 transfected with *Nlrp6-Flag* plasmid or not, and then exposed with 5 mM fructose as well as 100 μM gastrodin or not, (*n* = 4). (C) Western blot detection of cleaved Caspase 3 and cleaved Caspase 9 in MPC5 transfected with *Nlrp6-Flag* plasmid or not, and then exposed with 5 mM fructose as well as 100 μM gastrodin or not, (*n* = 4). (D) Flow cytometry analysis of activated Caspase 3 in MPC5 transfected with *Nlrp6-Flag* plasmid or not, and then exposed with 5 mM fructose as well as 100 μM gastrodin or not, (*n* = 4). (E) ROS levels were considered as the fluorescence intensity of fluorogenic probe DCFH2-DA, measured via microplate reader in MPC5. MPC5 were transfected with *Nlrp6-Flag* plasmid or not, and then exposed with 5 mM fructose as well as 100 μM gastrodin or not, (*n* = 8). (F) Mitochondrial ROS production was considered as the fluorescence intensity of labeling fluorogenic

probe MitoSOX, measured by flow cytometry in MPC5. MPC5 were transfected with *Nlrp6-Flag* plasmid or not, and then exposed with 5 mM fructose as well as 100 μM gastrodin or not, ($n = 4$). (G) OCR detection in MPC5 transfected with *Nlrp6-Flag* plasmid or not, and then exposed with 5 mM fructose as well as 100 μM gastrodin or not, ($n = 4$). (H) Flow cytometry analysis of mitochondrial membrane potentials using JC-10 dye in MPC5, which were transfected with *Nlrp6-Flag* plasmid or not, and stimulated with 5 mM fructose as well as 100 μM gastrodin or not, ($n = 3$). (I) Western blot detection of Cyto C performed on mitochondrial and cytosolic fractions in MPC5 transfected with *Nlrp6-Flag* plasmid or not, and then exposed with 5 mM fructose as well as 100 μM gastrodin or not, ($n = 3$). (J) Representative IF images of TUNEL assay in MPC5 transfected with *Nlrp6-Flag* plasmid or not, and then exposed with 5 mM fructose as well as 100 μM gastrodin or not, Scale: 100 μm. (K) Flow cytometry analysis of apoptotic cells through Annexin V-FITC/PI staining in MPC5, which were transfected with *Nlrp6-Flag* plasmid or not, and stimulated with 5 mM fructose as well as 100 μM gastrodin or not, ($n = 5$). Data are expressed as the mean ± SEM. $^{*}P < 0.05$, $^{**}P < 0.01$, $^{***}P < 0.001$.

Table S1. Sequence for siRNA transfection of podocytes.

Gene	Sequence
siRNA-*Nlrp6*	(F) CUCCGUGUCCGAGUACAAGdTdT
	(R) CUUGUACUCGGACACGGAGdTdT
siRNA-*Bok*	(F) GAGAUGAGCUGGAGCAGAUdTdT
	(R) AUCUGCUCCAGCUCAUCUCdTdT
siRNA-NC	(F) UUCUCCGAACGUGUCACGUdTdT
	(R) ACGUGACACGUUCGGAGAAdTdT

Table S2. Antibody list used in this study.

Antibodies	Source	Identifier
Anti-NLRP6	Sigma	SAB1302240
Anti-Synaptopodin	Santa Cruz	sc-515842
Anti-NPHS2(Podocin)	Abcam	ab50339
Anti-Nephrin	Abcam	ab58968
Anti-BOK	Abcam	ab186745
Anti-TRIM7	Biorbyt	orb1892
Anti-FAM213A	Sigma	HPA009025
Anti-Cytochrome C	Abcam	ab133504
Anti-BAK	CST	12105S
Anti-BAX	Santa Cruz	sc-7480
Anti-Bcl-2	Abcam	ab182858
Anti-COX IV	Abcam	ab33985
Anti-Caspase 3	Abcam	ab184787
Anti-cleaved Caspase 3	Abcam	ab214430
Anti-cleaved Caspase 9	Abcam	ab202068
Anti-Flag-Tag	Abcam	ab1162
Anti-HA-Tag	CST	3724S
Anti-IgG	CST	2729S
Anti-VDAC	Abcam	ab14734
Anti-β-actin	Zoonbio	ABM-001-100
Anti-TOMM20	Abcam	ab186735
HRP-conjugated Affinipure Goat Anti-Rabbit IgG(H+L)	Proteintech	SA00001-2
HRP-conjugated Affinipure Goat Anti-Mouse IgG(H+L)	Proteintech	SA00001-1
Alexa Fluor 488	Invitrogen	A-11008
Alexa Fluor 555	Invitrogen	A-21428
Alexa Fluor 647	Invitrogen	A-21424

Table S3. The primer list for qRT-PCR.

Gene	Sequence
Nlrp6	(F) GACGAGAGGAAGGCAGAG
	(R) TGGTGATGAAGAGCAGGT
Nlrp12	(F) AAGACCGCAATGCACGATTAG
	(R) TGGAGCGTTCCCACTCTACA
Bok	(F) AAGGTAGTGTCCCTGTATTCCG
	(R) AGGTCTTGCGTACAAACTCCC
Trim7	(F) ACAGAAACAGAATGAGAACCTGG
	(R) GCTCAGTGTGCTTTTGAACTCC
β-actin	(F) GTGACGTTGACATCCGTAAAGA
	(R) GCCGGACTCATCGTACTCC
